# Evolution of protein *N*-glycosylation process in Golgi apparatus which shapes diversity of protein *N*-glycan structures in plants, animals and fungi

**DOI:** 10.1038/srep40301

**Published:** 2017-01-11

**Authors:** Peng Wang, Hong Wang, Jiangtao Gai, Xiaoli Tian, Xiaoxiao Zhang, Yongzhi Lv, Yi Jian

**Affiliations:** 1 Tropical Crops Genetic Resources Institute, Chinese Academy of Tropical Agricultural Sciences & Ministry of Agriculture Key Laboratory of Crop Gene Resources and Germplasm Enhancement in Southern China, Danzhou, Hainan 571737, China; 2 Molecular Immunology and Antibody Engineering Center, College of Life Sciences, Jinan University, Guangzhou, Guangdong 510632, China; 3 Department of Anesthesia and Perioperative Care, University of California, San Francisco, San Francisco, CA 94143, USA; 4 State Key Laboratory of Desert and Oasis Ecology, Xinjiang Institute of Ecology and Geography, Chinese Academy of Sciences, Urumqi 830011, China

## Abstract

Protein *N*-glycosylation (PNG) is crucial for protein folding and enzymatic activities, and has remarkable diversity among eukaryotic species. Little is known of how unique PNG mechanisms arose and evolved in eukaryotes. Here we demonstrate a picture of onset and evolution of PNG components in Golgi apparatus that shaped diversity of eukaryotic protein *N*-glycan structures, with an emphasis on roles that domain emergence and combination played on PNG evolution. 23 domains were identified from 24 known PNG genes, most of which could be classified into a single clan, indicating a single evolutionary source for the majority of the genes. From 153 species, 4491 sequences containing the domains were retrieved, based on which we analyzed distribution of domains among eukaryotic species. Two domains in GnTV are restricted to specific eukaryotic domains, while 10 domains distribute not only in species where certain unique PNG reactions occur and thus genes harboring these domains are supoosed to be present, but in other ehkaryotic lineages. Notably, two domains harbored by β-1,3 galactosyltransferase, an essential enzyme in forming plant-specific Le^a^ structure, were present in separated genes in fungi and animals, suggesting its emergence as a result of domain shuffling.

Genes with new functions emerge continuously throughout the tree of life. A new gene arises within a specific phylogenetic lineage, which is not similar in sequence with any other genes in organisms that have split evolutionarily before this time^1^,^2^. In terms of how origin of gene novelties occurs, there are two major models: duplication-divergence model, which proposes an initial duplication of an existing gene followed by rapid divergence, and *de novo* evolution model, which assumes that a new gene emerges out of non-coding DNA^1^,^3^,^4^. Phylogenetic analyses suggested that *de novo* evolution of new genes occurred throughout evolutionary time, although non-coding DNA sources are not always identified for some claimed *de novo* genes^5^. Although less common, gene fusion, by which multiple transcription units merge into one compact unit, is an important source of new gene emergence^6^. Considering functional and structural significance of evolution, modularity of protein evolution plays a remarkable role in shaping the genomic make-up, which is generally associated with domains. Domains are functional constituents of proteins more conserved than other parts of genes in sequence, and are thus evolutionarily conserved across taxa. Genes arising by duplication-divergence is attributable enormously to domain duplication and divergence, and *de novo* gene births constantly give rise to new domains^7^. Likewise, gene fusion essentially occurs through recombination of conserved domains, namely domain shuffling, that are found to be present in different instances^8^,^9^.

In this article, we investigate how genes responsible for protein *N*-glycosylation (PNG) arose in eukaryotes. Particularly, we study the roles that domain emergence and combination as well as sequence divergence played in gene evolution in this process. Glycosylation is one of the most complex post-translation modifications of proteins, which is common for secretory proteins in eukaryotes^10^. *N*-linked glycans are widely observed to be crucial for proper folding of proteins, which provide blueprints for precise instruction of protein folding and discrimination signals for quality control systems^11^. Thus, it can be important for the function of individual glycoproteins, which would further have physiological effects on eukaryotic cells. Biological activities of many therapeutic proteins rely heavily on their glycosylation status. As a result, protein glycosylation is one of the main focuses in the biopharmaceutical research community^10^,^12^. Carbohydrates attached to the proteins can be classified into two categories, *N*-glycans, which are linked to the amide group of asparagine residues, and *O*-glycans, present on the hydroxyl group of serine, threonine, hydroxylysine and hydroxyproline residues^10^.

PNG is catalyzed with the form of a rational orchestration of multiple enzymatic formation and breakdown of glycan linkages, which is achieved by glycosyltransferases and glycosidases, occurring in the endoplasmic reticulum (ER) firstly and then in Golgi apparatus. Reaction mechanisms in ER are largely conserved in yeasts, mammals and plants. The mechanisms are initialized at the cytosolic side of the ER membrane by transferring an oligosaccharide precursor, Man_5_GlcNAc_2_, from a dolichol lipid carrier onto specific Asn residues constitutive of the consensus sequence Asn-X-Ser/Thr/Cys in nascent proteins^10^,^13^,^14^,^15^. Afterward, reactions proceed in ER lumen with complete assembly of the Glc_3_Man_9_GlcNAc_2_ precursor catalyzed by sequential orchestration of multiple enzymatic steps^10^,^12^,^16^.

While the assembly mechanism of core *N*-glycan precursor is conserved in ER, further modifications in Golgi apparatus vary enormously in different eukaryotic lineages, depending on a rich genetic toolbox of enzymes that are used to generate different types of *N*-glycans; how genes encoding these enzymes emerged and evolved is the focus of this article. In yeasts, a single α1,6 mannose unit is first attached to the glycan by Och1; then, it is elongated by multi-enzyme complexes, M-PolI and M-PolII, to form the α1,6 outer chain backbone containing up to 50 additional mannose residues. It is further decorated with side chains mainly consisting of homopolymeric α1,2 mannosides and heteropolymeric α1,2/α1,3 or α1,2/β1,2 mannosides, catalyzed by Mnn1, Mnn2, Mnn5 and Mnn6, respectively^17^,^18^. This machinery confers *N*-glycans distinguished properties of immense proportions of mannose redsidues in yeasts (Fig. 1a). In higher plants and mammals, however, *N*-glycans are made up by an enormously greater variety of monosaccharide residues (Fig. 1b and c)^10^,^19^,^20^. Firstly, the high-mannose *N*-glycan core is trimmed and acetylglucosamine is attached, which are catalyzed by β1,2 *N*-acetylglucosaminyltransferase I, Golgi α-mannosidase II and β1,2 *N*-acetylglucosaminyltransferase II sequentially, before it is further modified where they have remarkable differences in plants and animals. In plants, the glycan core is usually substituted by a β1,2 xylose, which is catalyzed by β1,2 xylosyltransferase (β1,2-XylT), and the proximal *N*-acetylglucosamine is replaced by an α1,3-fucose through catalysis of α1,3-fucosyltransferase (α1,3-FucT); also, in higher plants a typical *N*-glycan usually contains a Lewis a (Le^a^) structure, which is formed by attachment of β1,3 galactose and α1,4 fucose to the terminal GlcNAc, facilitated by β1,3-GalT and α1,4-FucT, respectively (Fig. 1b). In mammals, a β1,4-galactose, combined with a sialic acid, is often attached to GlcNAc residue, which is catalyzed by β1,4 galactosyltransferase and α2,6 sialyltransferase sequentially. Also, tri- and tetra-antennary branched complex *N*-glycans are common extensions in mammals, which are facilitated by GnTIII, GnTIV and GnTV, respectively (Fig. 1c).

Evolutionary origins of *N*-glycosylation occurring in ER have been demonstrated to be conserved among eukaryotic lineages^21^. We believe that diversity and novelties of PNG, and hence structures of *N*-glycans of proteins in different domains of eukaryotic lives, should be reflected by gene novelties coding for enzymes in the PNG pathways in Golgi. However, our knowledge of origin and evolution of PNG reactions in Golgi apparatus is dispatched. In this article, we aim to systematically investigate how molecular mechanisms of PNG in Golgi emerged and evolved, based on which we propose how they shaped diversity and novelty of protein *N*-glycans in different eukaryotic lineages. On the basis of identification of conserved domains in the PNG genes already characterized, we systematically identified genes containing the domains through combination of BLAST and HMMER, facilitated by whole genome sequencing and assembly data available. Based on the gene sequences retrieved, we sought to answer when novel mechanisms of PNG possibly occurred, and how they evolved in fungi, animals and plants.

## Results

### Reference sequence collection and domain identification

**Figure 1:**
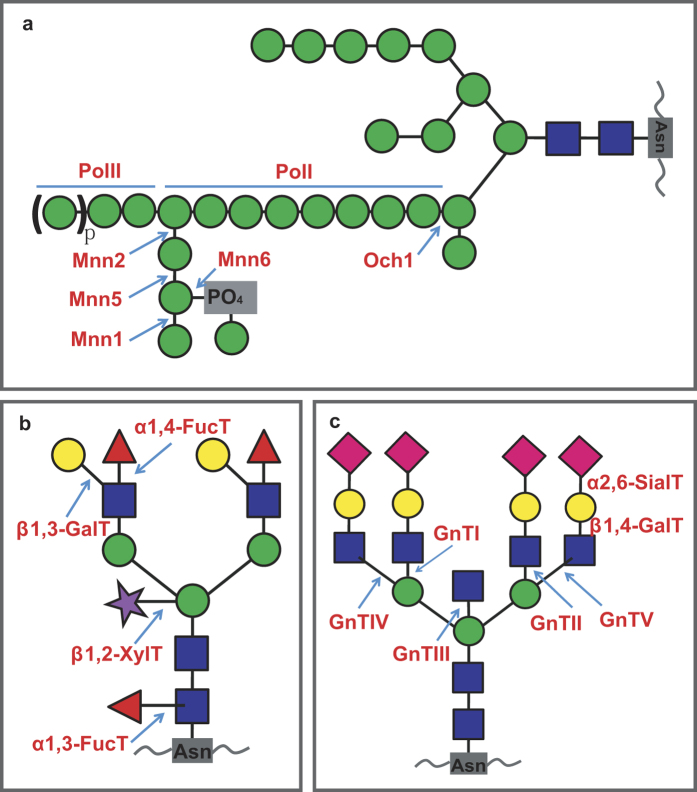
Typical structure of *N*-glycans of fungal (**a**), plant (**b**) and animal (**c**) proteins. The depiction is adapted from Gomord *et al*. and Castilho *et al*.^19^,^65^. The *N*-glycans are attached to contiguous asparagine residues with the consensus sequence Asn-X-Ser/Thr/Cys. Labels in red are enzymes for the steps involved in lineage-specific *N*-glycan modifications. Glycan residue representation for icons is shown at bottom.

Reference PNG gene sequences with known enzymatic functions were retrieved based on recent articles^10^,^12^,^16^,^19^,^22^. In total, 11 sequences from *Saccharomyces cerevisiae*, 8 from *Arabidopsis thaliana*, and 5 from *Homo sapiens* were collected (Table 1, Fig. 1, Supplementary Table S1). Of the 24 genes, all encode glycosyltranseferases, except for two mannosidases (MNS1 and GMII). 23 Pfam domains were identified in the peptide sequences of the 24 genes. Among them, combination of domains were identified in Och1, Van1, α1,4-FucT, and GnTV, respectively, while several domains were shared by multiple (2–3) enzymes (Table 1).

Interestingly, 9 domains, which were embodied in sequences encoding glycosyltransferases, belonged to the same clan (Pfam ID: CL0110) (Table 1). Based on Pfam definition, a clan contains multiple Pfam families that have descendent from a single evolutionary origin^23^. In the Pfam database, the clan CL0110 contains 46 families of glycosyltransferases possessing a Rossmann-like fold structure^24^. In total, domains in 15 out of 22 glycosyltransferase genes in PNG could be classified into the clan CL0110, suggesting that these genes have risen from a single evolutionary origin^23^. Notably, all domains in genes responsible for fungus-specific modifications belong to this clan, suggesting that the metabolic pathway leading to the branched structures with dense mannosylation took shape by duplication and divergence of a single sequence evolutionarily in fungi (Fig. 1, Table 1).

### Identification of PNG genes by domain recognition

Based on the reference peptide sequences and the identified domains, we sought a comprehensive identification from selected sequenced genomes. We chose species of representative taxa in the tree of life with high-quality genome assembly data publicly available. Peptide gene model files of 15 Archaea and 52 Bacteria were downloaded, whose genomes are completely assembled, and those of 24 fungal species and 34 animal species were obtained, whose chromosome-level genome assembly data are available. Gene model files of 28 plant species were downloaded, among which genomes of 21 species are assembled to chromosome-level, and 7 are assembled to scaffold-level whose taxa represent algae, lower vascular plants, and *Amborella* which is close to the base of the flowering plant lineage. Hence, in total, we used 153 genomes for identification of PNG domain-containing genes (Supplementary Table S2).

**Figure 2:**
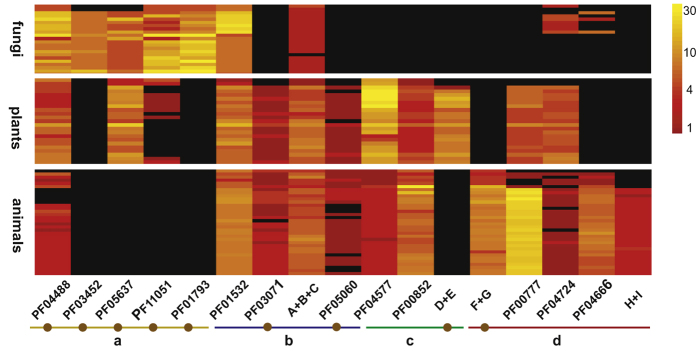
Presence and abundance of genes containing domains related to PNG in eukaryotes. The heat map depicts values of 10 of the logarithm of absolute gene number counts for each genome. Black represents no sequences identified for the gene in the genome. Brown dot below the Pfam domain IDs indicates that the domains are classified under the clan CL0110. A, PF01074; B, PF09261; C, PF07748; D, PF00337; E, PF01762; F, PF13733; G, PF02709; H, PF15027; I, PF15024. (**a**) Genes containing the domains related to PNG are only present in fungi; (**b**) genes related to PNG are only in plants and animals; (**c**) only in plants; (**d**) only in animals.

Based on the identified domains in reference genes of PNG enzymes, we used HMMER, a domain-centric method to compare profile hidden Markov models (HMMs) of PfamA to peptide datasets, to identify homologous sequences, by which 4491 sequences were obtained in total^25^,^26^. BLAST searches were performed too, which did not generate any sequences beyond HMMER search results. 6 domains or domain combinations as contained in PNG sequences are confined in a specific eukaryotic lineage; this distribution is consistent with that of genes containing these domains related to PNG (Fig. 2, Supplementary Figure S1). For example, Mnn9, Van1 and Anp1, which are involved in protein *N*-mannosylation in fungi, are supposed to be present in fungi; all these genes contain the same domain PF03452, and genes with this domain were only identified in fungi^18^,^27^,^28^. Likewise, activity of β1,4-GalT was only identified in animals, and the peptide sequence contains domains PF13733 and PF02709; genes containing both the domains were only identified in animals^29^,^30^. Although some genes are only present in specific lineages, sequences containing the domains in these genes were identified in other lineages too. For example, β1,2-XylT is only present in plants, which has the domain PF04577, but genes containing this domain were identified in animals as well as in plants (Fig. 2)^31^. Generally, if the genes containing the domains are present in kingdoms where a specific PNG reaction is not supposed to occur, genes would be remarkably more abundant in kingdoms where genes responsible for specific PNG mechanisms are present, except domains like PF05637 and PF00852 (Fig. 2).

### Gene evolution in fungal *N*-mannosylation

Fungal glycosylation is characterized by dense mannosylation which mainly takes place in Golgi apparatus. 11 enzymes were characterized to be involved in fungal Golgi *N*-mannosylation. Peptide sequences of these enzymes were classified into 5 pfam families, and could be further assigned to the same clan, CL0110, which indicates that the glycosylation enzymes in sophisticated fungal PNG mechanism could be traced back to a single source evolutionarily, which probably occur by gene duplication and divergence.

While genes containing PF03452 and PF01793 were only identified in fungi, PF05637 and PF11051 were identified in plants as well, and PF04488 were identified in plants and animals as well as in fungi.

Mnn9 and Van1 make up mannan polymerase I (Man Pol-I), and Anp1 is a component of mannan polymerase II (Man Pol-II)^18^,^27^,^28^. All these 3 enzymes belong to the family PF03452. Interestingly, PF03452 genes were identified only in Saccharomyceta; over 3 copies of genes were present in each genome of these species. In phylogeny, these genes were classified into 3 major clades (Supplementary Figure S1). Genes in Clade II were split into 2 minor groups in Saccharomucetales (Clades IIa and IIb, Supplementary Figure S1). Only leotiomyceta genes were identified in Clade III. Mnn6 contain domain PF01793, which was clustered in Clade I of the 3 major clades of the phylogeny tree of PF01793, as shown in Supplementary Figure S2.

Both Mnn10 and Mnn11 contain domain PF05637. These genes were shown to be present not only in fungi but in plants. In *E. cuniculi* and Basidiomycota representing basal fungi clades, no homologs were identified. The phylogeny of this family resolved multiple distinctive clades, which indicated that the members have evolved into different biological/enzymatic roles. Mnn10 and Mnn11 represented the only 2 members of PF05637 in *S. cerevisiae*, which are in Clade I and IV, respectively. Only plant members are represented in Clade II. In Clade III, only leotiomyceta homologs were included, while in Clade V, there are leotiomyceta and plant homologs (Supplementary Figure S3). Indeed, the plant homologs have shown to not play roles in glycoprotein biosynthesis, but are involved in plant cell wall biosynthesis^32^,^33^.

6 *S. cerevisiae* genes contain domain PF11051. Among them, MNN2 (YBR015C) and MNN5 (YJL186W) are specifically involved in *N*-glycan formation, which are responsible for the addition of the first and second α1,2-linked mannose, respectively, to form the branches on the mannan backbone of oligosaccharides^34^. MNN1 are involved in both *N*- and *O*-glycosylation, while the other 3 are specifically involved in *O*-glycosylation^35^,^36^. The phylogeny partitioned the PF11051 members into 2 groups largely, in which MNN2 and MNN5 are placed in the same group (Clade I), and the other 4 *S. cerevisiae* genes are in the other group (Clade II) (Supplementary Figure S4). An Arabidopsis gene was identified, which was grouped in the clade II, whose biological function has not been reported yet, to our knowledge (Supplementary Figure S4).

Och1 (YGL038C) initiates *N*-mannosylation in Golgi by attaching an α1,6 mannose unit to the oligosaccharide core^37^. Hoc1 (YJR075W) is a component of M-PolII which adds α1,6 mannose residues to the core^38^,^39^. Both peptides were identified to contain PF04488 domain. Besides these genes, 2 more were identified from *S. cerevisiae*, which have been characterized to play as Mannosylinositol phosphorylceramide (MIPC) synthase catalytic subunits, and be involved in sphingolipid biosynthesis^40^; these genes form a single clade in phylogeny, in which plant and animal genes are included too (Clade II) (Supplementary Figure S5).

### Gene Evolution of PNG enzymes shared by plants and animals

In Golgi apparatus, several α1,2-linked mannose residues need to be removed to provide the Man_5_GlcNAc_2_ substrate for the formation of complex *N*-glycans in animals and plants. In human, this reaction is catalyzed by 3 isoforms of Golgi-α-mannosidase^41^. In Arabidopsis, 2 isoforms of this enzyme are present in the genome^42^,^43^. These enzymes belong to class I α-mannosidases, which harbor a conserved domain PF01532. A thorough retrieval of sequences containing PF01532 was conducted, and the results indicated that genes in this family are present not only in plants and animals, but also in fungi. Phylogeny inference, however, indicated that plant and animal genes encoding Golgi-α-mannosidases were in 2 clusters close to each other (Supplementary Figure S6, Clades I and II). In these clusters, no fungi genes were contained. The clade close to Clades I and II comprises of fungal genes as well as animal and plant genes (Supplementary Figure S6, Clade III). In this clade, α-mannosidases from yeast (YJR131W), Arabidopsis (AT1G30000) and human (ENSG00000177239.14) are included, which all have been demonstrated to reside in ER and involved in ER-associated degradation (ERAD) of misfolded glycoproteins^44^,^45^,^46^,^47^. This indicates genes in the clade close to Golgi-α-mannosidase clade encode ER-associated α-mannosidases involved in protein quality control. Clade IV only contains fungal genes; in this clade, a yeast gene encoding mannosidase (YLR057W) is comprised, which was proved to be a novel component of ERAD pathway. Clade V is distant to any other clades, comprises genes in all three kingdoms (Supplementary Figure S6). In this clade, three human genes encode enzymes playing roles as ER degradation enhancers (EDEMs, ENSG00000134109, ENSG00000088298 and ENSG00000116406)^48^,^49^,^50^. Also, the yeast and Arabidopsis genes are involved in ERAD^46^,^51^. These results indicate that genes in all the clades are involved in ERAD, except genes in Clades I and II encoding Golgi-α-mannosidases, which only contain genes from animals and plants.

In animals and plants, β1-2-GlcNAc by GlcNAc transferase I (GnTI) starts the diversification of Man5GlcNAc2^52^. In both Arabidopsis and human, a single gene is responsible for this role (AT4G38240 and ENSG00000131446, respectively)^53^,^54^,^55^. All these genes contain the domain PF03071. Genes were only identified in animals and plants. Phylogenetic analyses resolved four major clades. Plant genes are all in a single clade (Supplementary Figure S7, Clade I), and Chordata genes containing human GnTI gene (ENSG00000131446) was in another single clade (Supplementary Figure S7, Clade II). 3 *C. elegans* genes encoding GnTI were in an independent clade (Supplementary Figure S7, Clade III)^56^,^57^. The Clade IV, as shown in Supplementary Figure S7, comprising Chordata genes, contains a human gene encoding protein *O*-linked mannose *N*-acetylglucosaminyltransferase (ENSG00000085998); alterations of this gene have been shown to cause muscle-eye-brain disease and several congenital muscular dystrophies^58^,^59^.

Following the addition of a β1,2-GlcNAc by GnTI, α1,3- and α1,6-Man were removed from the core *N*-glycan substrate by α-mannosidase II (GMII)^10^. In human genome, 2 genes code for this enzyme^60^,^61^. Peptide sequences of these genes contain 3 domains: PF01074, PF09261 and PF07748. Genes harboring all the domains are identified in fungi as well as in animals and plants. However, as shown in phylogenetic tree, only plant and animal genes were present in a major clade, in which no genes were identified in fungi, indicating that genes encoding GMIIs are not present in fungi (Supplementary Figure S8, Clade I). The clade close to the group GMII contains genes encoding vacuolar α-mannosidases (YGL156w in yeast, and ENSG00000140400 in human, as shown in Supplementary Figure S8, Clade II)^62^,^63^. In another major clade, genes were only present in plants and animals, which likely encode α-mannosidases hydrolyzing terminal non-reducing α-D-mannose residues^64^,^65^.

Golgi β1,2-*N*-acetylglucosaminyltransferase II (GnTII) catalyzes the conversion from hybrid to complex *N*-glycans^10^. Peptide sequences of this gene contain a domain PF05060, and sequences of this enzyme were only identified in plants and animals; in each species, only 1–2 copies of genes were present in the genome.

### Evolution of genes encoding PNG machinery specific for animals

β-1,4-galactosyltransferases (β1,4-GalT) form a family with seven members, which all have exclusive specificity for the donor substrate UDP-Gal, and all transfer Gal in β-1,4 linkage to GlcNac, Glc and Xyl^29^. One of them, β1,4-GalT I, catalyzes attachment of β1,4-galactose to GlcNAc residue, which is absent in plants and fungi^30^,^66^. In humans, this enzyme is encoded by ENSG00000086062^67^,^68^. The peptide sequence of this enzyme contains 2 Pfam domains (Domain IDs: PF13733 and PF02709). Genes containing these domains were identified in animals, while no genes were identified in fungi and plants. In phylogeny, the genes closest to the clade containing ENSG00000086062 likely encoded β1,4-GalT II which synthesizes *N*-acetyllactosamine in glycolipids and glycoproteins (Supplementary Figure S9)^69^,^70^.

Another animal-specific glycosyltransferase is α-2,6 sialyltransferase (α2,6-SialT), which catalyzes the transfer of sialic acid residue to terminal nonreducing positions of oligosaccharide chains of glycoproteins^71^. In humans, this peptide is encoded by gene ENSG00000117069, which is a Type II membrane protein and belongs to a family with multiple members^71^,^72^,^73^. Every known peptide sequence of these proteins was identified to harbor domain PF00777. Phylogenetic analyses of the sequences containing this domain showed that animal α2,6-SialT genes formed a monophyletic group (Supplementary Figure S10). Interestingly, plant homologs were present, which were placed close to the animal α2,6-SialT genes in the phylogenetic tree (Supplementary Figure S10). In plants, no sialic acid has been detected^74^. 2 Arabidopsis SiaT-like genes were suggested to be involved in transfer of 2-keto-3-deoxylyxo-heptulosaric acid and 2-keto-3-deoxymanno-octulosonic acid to Rhamnogalacturonan-II in pectic polymer biosynthesis and to be required for proper pollen tube elongation^75^.

Formation of tri- and tetra-antenary complex *N*-glycans are common in mammalian glycoprotein modification, while plant and fungal glycoproteins lack these multiantenary glycans^10^,^76^. These branched structures are associated with various physiological processes such as cancer metastasis and T-cell activation, and the glycans influence protein propertities including immunogenicity, stability and pharmacokinetics^20^,^77^,^78^. Branching of these *N*-glycans are catalyzed by several acetylglucosaminyltransferases (GnTIII, GnTIV and GnTV, respectively)^79^. GnTIII catalyzes the addition of *N*-acetylglucosamine in β1-4 linkage to the β-linked mannose of the trimannosyl core of *N*-linked sugar chains to produce a bisecting GlcNAc residue^80^. Domain PF04724 was identified in human GnTIII peptide sequences. Genes containing this domain were identified in animals as well as in plants and fungi; in most of the animal genomes, only 1–2 copies of the genes were present, while multiple copies of the genes were identified in every genome of the plants we investigated. Phylogenetic topology of animal genes in this family largely conformed with animal taxonomy (Supplementary Figure S11). In the phylogenetic tree, plant homologs were clustered together, none of which have been functionally characterized, to our knowledge. However, reports suggested that some genes were possibly involved in pollen germination and pollen tube development (Supplementary Figure S11)^81^. GnTIV catalyzes the transfer of GlcNAc from UDP-GlcNAc in β1-4 linkage to α1,3-D-mannoside on GlcNAcβ1-2Manα1-6(GlcNAcβ1-2Manα1-3)Manβ1-4 GlcNAcβ1-4GlcNAcβ1-Asn^82^,^83^. The peptide sequences of this gene were identified to contain domain PF04666. 4 homologs of GnTIV were present in human genome^84^,^85^. No genes were identified in land plants, while the genes were widely present in animal genomes. Phylogenetic inference showed that the four GnTIV genes were clustered into two major clades, with GnTIVA and GnTIVB, the two function-characterized genes clustered in Clade I as shown in Supplementary Figure S12. GnTV transfers *N*-acetylglucosamine (GlcNAc) to the C-6 position of the α1,6-linked mannosyl residue in the trimannosyl core structure of complex *N*-glycans to generate GlcNAc(α1,6) mannose^86^. 2 isoforms of GnTV gene are present in the human genome^87^,^88^. Two domains, namely PF05027 and PF15024, were identified in peptide sequences of GnTV. Genes harboring PF15027 were only identified in animals, while genes harboring PF15024 were identified in some dicotyledonous plants, ferns, algae as well as in animals. These domains could not be classified into any clans, and no reports indicate genes containing these domains in other eukaryotic kingdoms have any other activities, thus allowing us to conclude that this gene emerged *de novo*. Phylogeny of genes with the domain PF15024 showed that the animal genes were mostly split into 2 clades, with most species we studied in this article represented in each clade. This shows that animal GnTV experienced duplications in early stage of animal evolution, with genes in both clades retained in animal genomes (Supplementary Figure S13). In the phylogenetic tree, plant genes harboring domain PF15024 were present in a monophyletic clade; the plant genes have lost the domain PF15027, suggesting loss of GnTV activities with these genes (Supplementary Figure S13).

### Evolution of PNG Genes specific for plants

In plants, complex-type *N*-glycans are structurally unique. β-mannose of the glycan core is attached by a bisecting β1,2-xylose, and proximal *N*-acetylglucosamine of the glycan core is substituted by an α1,3-fucose. Also, β1,3 galactose and α1,4-fucose link to the terminal GlcNAc of *N*-glycans, which form the Lewis a (Le^a^) oligosaccharide structure. These unique characteristics of the *N*-glycan structure are believed to be conferred by the plant-specific components of the enzymatic machinery in plants. Progresses have been made to identify the genes responsible for the formation of these structures in model plant Arabidopsis^31^. However, few reports were available on the genes in these steps in other plant species, whose origins and evolutions are still unknown, to our knowledge.

β1,2-xylosyltransferase (XylT) catalyzes the transfer of xylose to the *N*-glycans in glycoproteins in plants. Only the gene in Arabidopsis was enzymatically identified to be XylT *in vitro* and *in vivo*, despite some reports of enzyme purification of XylTs from other plants^31^,^89^,^90^. Domain PF04577 was identified in Arabidopsis XylT peptide sequence (AT5G55500). Genes containing the domain PF04577 were not identified in fungi. Only 1–3 genes were present in each animal genome, while in plants, genes are abundant (as many as over 30 copies in each genome). Phylogenetic analyses resolved the gene family into 3 major groups, and Group I could be further split to 3 clades, namely Clades Ia, Ib and Ic, respectively (Supplementary Figure S14). Arabidopsis XylT gene was placed under the Clade Ib in Group I; in this clade, only plant genes were present, indicating that genes in this clade represent XylT genes in plants. Clade Ia contains animal and plant genes; in this clade, no plant genes have been enzymatically or physiologically characterized, but the human gene ENSG00000144647 was identified to encode a protein *O*-linked mannose *N*-acetylglucosaminyltransferase, suggesting roles of the genes in this clade involved in protein *O*-glycans^91^,^92^. In Clade Ic, animal genes are grouped together, among which human gene ensg00000163378 was identified as a EGF domain specific *O*-linked *N*-acetylglucosamine transferase^93^. Genes in Groups II and III are all derived from plant genomes: Group II genes consist of *Klebsormidium flaccidum* genes, while genes in Group III consist of genes from species spanning from lower to higher land plants (Supplementary Figure S14). In this group, although no genes were definitely identified enzymatically, AT3G10320 was demonstrated as a putative xylosyltransferase which was recently characterized as MUCI21, while the genes AT3G18170 and AT3G18180 are expressed highly in a heteroxylan containing mucilaginous tissues, which indicated that the genes in this group are related to mucilage production in terrestrial plants^94^,^95^.

α1,3-fucose transferase (α1,3-FucT) and α1,4-fucose transferase (α1,4-FucT) add fucose residue to the basal and terminal part of the glycan core, respectively. In Arabidopsis, the genes encoding these enzymes were identified (AT3G19280 and AT1G49710 for α1,3-FucT, and AT1G71990 for α1,4-FucT)^96^,^97^. The Arabidopsis genes encoding both the enzymes conferred domain PF00852. Genes containing this domain were identified in both plants and animals, but not in other eukaryotic species. Copy numbers in animals are slightly more than those in plants. Phylogenetic analysis results showed that the plant sequences were clustered into two groups, and each contained one of Arabidopsis fucose transferases, respectively, indicating that the genes in these two groups represent genes encoding α1,3-FucT and α1,4-FucT, respectively, which play roles in plant-specific *N*-glycan modifications (Supplementary Figure S15, Clades I and II). In animal-specific Clades III, IV and V, genes likely code for fucoses too, whose substrates include polysaccharides and sphingolipids, and mutations in these genes have been demonstrated to be associated with a variety of human diseases^98^,^99^,^100^,^101^,^102^.

**Figure 3:**
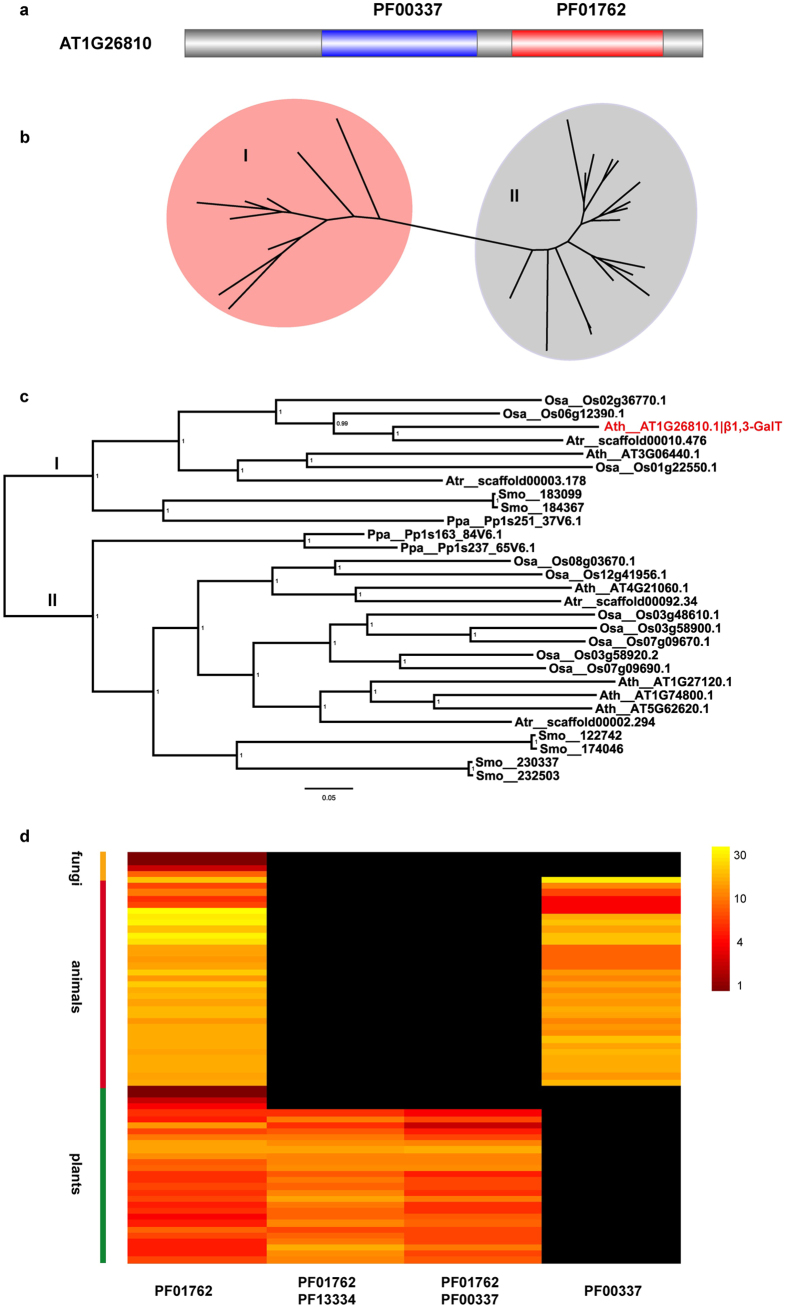
Evolution of β1,3-GalT containing domain PF01762 in eukaryotes. (**a**) Linear structure of Arabidopsis β1,3-GalT (AT1G26810), highlighting the two domains it contains. (**b**) Unrooted tipless phylogenetic tree of plant genes containing both domains PF01762 and PF00337, which shows that genes could be classified into two major groups. Group I is shaded in red, which includes β1,3-GalT, and the Group II is shaded in grey. (**c**) Phylogenetic tree with tip labels of plant genes containing both domains PF01762 and PF00337, in which the known Arabidopsis β1,3-GalT gene (AT1G26810.1, labeled in red) is placed in group I. (**d**) Abundance of genes containing domains PF01762, PF00337 and PF13334, respectively. The heat map depicts values of 10 of the logarithm of absolute gene number counts for each genome. Black shade represents no gene identified for the gene in a specific genome.

The other component of Le^a^ structure, β1,3-galactose, is attached by β-1,3 galactosyltransferase (β1,3-GalT). In Arabidopsis, this enzyme is encoded by AT1G26810^103^. The peptide sequence of this gene was identified to contain 2 Pfam domains, PF01762 and PF00337 (Fig. 3a). The sequences containing both these domains were only identified in land plants. There were 6 β1,3-GalT genes in Arabidopsis, consistent with Strasser *et al*.^103^. Phylogenetic analysis indicated that the genes split into 2 groups shortly after the origination of the genes containing the 2 domains (Fig. 3b). Strasser *et al*. posited that only 1 out of the 6 genes has β1,3-galactose activity^103^. In the phylogeny tree, this gene was clustered in Clade I, forming a monophyletic clade (Fig. 3b). Interestingly, both the domains PF01762 and PF00337 are present in animal genomes, but they were contained in separated genes (Fig. 3d; Supplementary Figure S16). The animal genes harboring the domain PF01762 include type II membrane-bound glycoproteins with diverse enzymatic functions which use different donor substrates including UDP-galactose and UDP-*N*-acetylglucosamine, and different acceptor sugars such as *N*-acetylglucosamine, galactose and *N*-acetylgalactosamine (Supplementary Figure S16)^104^. Genes containing the PF00337 domain are a gene family coding for β-galactoside-binding proteins, which are implicated in modulating cell-cell and cell-matrix interactions^105^,^106^. In algae, genes were identified containing only domain PF01762. In land plants, a group of genes were identified containing two domains, PF01762 and PF13334; PF13334 emerged in land plants, and were not identified in any other eukaryotic lineages. This group of genes contained *O*-galactosyltransferases involved in cell wall formation and embryo development (Supplementary Figure S16)^107^,^108^. Our results indicated that the β-1,3 galactosyltransferase genes, which are essential in construction of Le^a^ structure in land plants, originated by combination of two domains, PF01762 and PF00337, followed by sequence duplication and divergence of new genes.

## Discussion

Structural diversity of protein *N*-glycans is believed to be attributable to differences of protein *N*-glycosylation (PNG) mechanisms, which largely is confined to Golgi apparatus, among eukaryotic lineages. A recent work has been performed to characterize origin and early evolution of PNG machinery in ER, which is largely consistent among fungi, plants and animals^21^. Although extensive studies have been made to characterize genes constituting unique PNG machinery components, little is known of how the genes expressed in Golgi apparatus emerged and evolved in eukaryotes. With the availability of high-quality genome assembly data, we carried out a comprehensive identification of PNG genes in Golgi, and studied evolution of the genes with the emphasis on evolution of domains^25^. A domain is a conserved region which is more conserved than other regions in a gene. We believe that domain-centric approaches could result in much more comprehensive identification of interested genes, as domains are conserved regions which are much more slowly diverged in sequence of evolution in the tree of life. Homology-based approaches like BLAST rely heavily on parameters such as e-value, which may introduce subjectivity. Hence, besides identification of homologs of PNG genes using BLAST, we carried out comprehensive survey of genes containing the domains that the PNG genes harbor in genomes of high quality which represent major species from archaea, bacteria, fungi, animals and plants through HMMER. In total, we obtained 4491 genes from 153 genomes, of which most were identified in eukaryotes. Further, we conducted phylogenetic analyses, together with extensive literature investigation, to help us to infer fucntions of the genes. This way, we believe it offers basis to provide fuller picture of eukaryotic PNG machinery evolution than by only retrieve of homologs of known genes.

24 domains were identified in the 23 known PNG genes. In yeast, 11 known PNG genes contained 5 conserved domains, which could be further classified into the same clan, CL0110. Also, GnTI and GnTII, the two glycosyltransferases shared by plants and animals, together with β1,3-GalT in plants and β1,4-GalT in animals could be classified into the clan CL0110, too. In total, 17 out of the 22 glycosyltransferase genes in PNG could be classified into the same clan. This clearly suggests single evolutionary source of the majority of genes constituting the complex and divergent PNG machinery. Although some genes, such as β1,2-XylT and α1,3-FucT in plants, and α2,6-SialT in animals, have kingdom-specific presence, genes containing domains that these genes contain could have genes identified in other kingdoms, indicating that emergence of these genes followed gene duplication and divergence model. Of all the 23 genes, only GnTV in animals, which harbors two domains, cannot be traced to any source, which indicates that this gene emerged *de novo* in animal lineage. Although less acknowledged, domain shuffling is an important way of new genes arising. In this study, β1,3-GalT is an example of domain shuffling, which takes an essential role in plant-specific Le^a^ formation. Peptide sequence of β1,3-GalT contains two domains, PF00337 and PF01762. Genes containing PF01762 were identified in fungi and animals too, and PF00337 was also present in animals. In animals, the two domains were in separated genes, and only in plants the two domains were identified to be fused, probably through domain shuffling.

Overall, this is an example that shows component novelty, which shapes uniqueness of a pathway in a lineage, could be achieved by varied evolutionary mechanisms. In this article, we showed that PNG genes in Golgi evolved mainly by duplication and further fast divergence, but there are cases of *de novo* evolution and domain shuffling in shaping novelty of PNG pathways in animals and plants. We believe that the protein *N*-glycosylation pathway is still fast evolving, and there should be unknown PNG elements awaiting identification. Our work provides foundation for further characterization of PNG mechanisms in Eukaryotes, and the results may have important implications for our understanding of evolution of genetic novelties shaping uniqueness of PNG pathways in Eukaryotes.

## Methods

### Sequence and domain identification

Reference peptide sequences were retrieved from Saccharomyces Genome Database (*S. cerevisiae*), TAIR (*A. thaliana*) and Ensembl Genomes (*H. sapiens*), respectively^109^,^110^. Pfam domain IDs were retrieved using pfam_scan.pl and PfamA database^111^,^112^. Carbohydrate-active enzyme categorization for reference genes were fulfilled with sequence-based annotation tool using CAZy Database through both BLAST and HMMER approaches using default values^113^,^114^.

Gene model files of 15 Archaea, 52 Bacteria, 24 fungal, 34 animal and 28 plant genomes were obtained, sources of which are recorded in Supplementary Table S2. The files were cleaned to only contain locus IDs in comment line, and removed symbols other than Roman letters in sequence lines which would otherwise interfere with gene identification and sequence analyses. Standalone BLAST searches were performed against the gene model files, using BLASTp in BLAST + suite, using reference peptide sequences obtained involved in protein *N*-glycosylation as queries, with E-values 1e-3^115^. Also, HMM searches of the 153 gene model files against the 23 domains in genes related to protein *N*-glycosylation, plus PF13334, which is fused with PF01762, were conducted respectively, with the “trusted cutoff” of the domains established by Pfam-A (ftp://sanger.ac.uk/) as the threshold for detecting the domain. Combination of BLAST and HMMER search results resulted in raw data files for each domain. Searches against Pfam-A family database using perl script pfam_scan.pl using “trusted cutoff” as the threshold, deletion of repeated sequences with 100% homology and visual inspection resulted in final versions of sequence data, with 4491 sequences obtained in total.

### Bioinformatic analyses

Sequences were analyzed by Probcons v1.12, and the alignments were visualized by BioEdit 7.2.5 ((http://www.mbio.ncsu.edu/)^116^. If needed, the alignment results were converted between from FASTA to NEXUS and/or PHYLIP formats. Bayesian phylogenetic analyses were conducted using MrBayes 3.1.2, with four Markov chains and two runs, with parameters set as default unless otherwise mentioned^117^. Standard deviation of split frequencies was checked after each 1,000,000 generations of each run to make sure they are below 0.05. The trees generated were visualized using Figtree v1.4.2 (http://tree.bio.ed.ac.uk/).

Gene structure was drawn using IBS 1.0.1, based on domain information retrieved from Pfam domain identification^118^. Gene number counts were converted to 10 of logarithmic values before heatmap illustration using pheatmap, an R package (https://cran.r-project.org/).

### Nomenclature

“Seven-kingdom system” developed by Michael *et al*. was used for classification of living organisms, in which the division of empire Prokaryota was introduced into two kingdoms, Bacteria and Archaea, and the empire Eukaryota was divided into five kingdoms, Protozoa, Chromista, Plantae, Fungi and Animalia^119^. We focus on three eukaryotic kingdoms, fungi, animals (Animalia) and plants (Plantae).

Gene family whose member containing *N*-glycosylation domain was named after the domain ID. For example, the gene family containing a domain PF01532, which encode mannosidases, is named PF01532 family. Unless otherwise indicated, genes were named after locus IDs, which were prefixed by abbreviations of species names and double underlines. For example, Arabidopsis thaliana gene AT1G26810 was labeled as Ath__AT1G26810. Human gene nomenclature was based on HUGO Gene Nomenclature Committee (HGNC) Database^120^,^121^.

## Additional Information

**How to cite this article**: Wang, P. *et al*. Evolution of protein *N*-glycosylation process in Golgi apparatus which shapes diversity of protein *N*-glycan structures in plants, animals and fungi. *Sci. Rep.* **7**, 40301; doi: 10.1038/srep40301 (2017).

**Publisher's note:** Springer Nature remains neutral with regard to jurisdictional claims in published maps and institutional affiliations.

## Supplementary Material

Supplementary Information

## Figures and Tables

**Table 1:** Known enzymes and identified domains responsible for PNG reactions.

	Gene	Species	Locus ID	CAZy Family	Domains	Clan
fungi	Och1	*S. cerevisiae*	YGL038C	GT32	PF04488	CL0110
	Mnn9	*S. cerevisiae*	YPL050C	GT62	PF03452	CL0110
	Van1	*S. cerevisiae*	YML115C	GT62	PF03452	CL0110
	Anp1	*S. cerevisiae*	YEL036C	GT62	PF03452	CL0110
	Mnn10	*S. cerevisiae*	YDR245W	GT34	PF05637	CL0110
	Mnn11	*S. cerevisiae*	YJL183W	GT34	PF05637	CL0110
	Hoc1	*S. cerevisiae*	YJR075W	GT32	PF04488	CL0110
	Mnn2	*S. cerevisiae*	YBR015C	GT71	PF11051	CL0110
	Mnn5	*S. cerevisiae*	YJL186W	GT71	PF11051	CL0110
	Mnn1	*S. cerevisiae*	YER001W	GT71	PF11051	CL0110
	Mnn6	*S. cerevisiae*	YPL053C	GT15	PF01793	CL0110
plants & animals	MNS1	*A. thaliana*	AT1G51590	GH47	PF01532	CL0059
	GnTI	*A. thaliana*	AT4G38240	GT13	PF03071	CL0110
	GMII	*A. thaliana*	AT5G14950	GH38	PF01074 PF09261 PF07748	CL0158 n/a CL0103
	GnTII	*A. thaliana*	AT2G05320	GT16	PF05060	CL0110
plants	β1,2-XylT	*A. thaliana*	AT5G55500	GT61	PF04577	n/a
	α1,3-FucT	*A. thaliana*	AT3G19280	GT10	PF00852	n/a
	β1,3-GalT	*A. thaliana*	AT1G26810	GT31	PF00337 PF01762	CL0004 CL0110
	α1,4-FucT	*A. thaliana*	AT1G71990	GT10	PF00852	n/a
animals	β1,4-GalT	*H. sapiens*	ENSG00000086062	GT7	PF13733 PF02709	CL0110 CL0110
	α2,6-SialT	*H. sapiens*	ENSG00000117069	GT29	PF00777	n/a
	GnTIII	*H. sapiens*	ENSG00000128268	GT17	PF04724	n/a
	GnTIV	*H. sapiens*	ENSG00000071073	GT54	PF04666	n/a
	GnTV	*H. sapiens*	ENSG00000152127	GT18	PF15027 PF15024	n/a

n/a, not available. Schematic representation of specific step for each enzyme is shown in Fig. 1. Sequences of the enzymes are in appendix S1.
