# Supplementary material for: Evolution of protein N-glycosylation process in Golgi apparatus which shapes diversity of protein N-glycan structures in plants, animals and fungi
Source: Sci Rep. 2017 Jan 11;7:40301. doi: 10.1038/srep40301 (PMC5225481; doi:10.1038/srep40301)
Supplement: Supplementary Information [file srep40301-s1.pdf]

Supplementary Information

Evolution of protein *N*-glycosylation process in Golgi apparatus which shapes diversity of protein *N*-glycan structures in plants, animals and fungi

Peng Wang[1,*], Hong Wang[2], Jiangtao Gai[1], Xiaoli Tian[3], Xiaoxiao Zhang[4], Yongzhi Lv[1], Yi Jian[1]

[1]Tropical Crops Genetic Resources Institute, Chinese Academy of Tropical Agricultural Sciences & Ministry of Agriculture Key Laboratory of Crop Gene Resources and Germplasm Enhancement in Southern China, Danzhou, Hainan 571737, China

[2]Molecular Immunology and Antibody Engineering Center, College of Life Sciences, Jinan University, Guangzhou, Guangdong 510632, China

[3]Department of Anesthesia and Perioperative Care, University of California, San Francisco, San Francisco, CA 94143, USA

[4]State Key Laboratory of Desert and Oasis Ecology, Xinjiang Institute of Ecology and Geography, Chinese Academy of Sciences, Urumqi 830011, China

[*]Correspondence: Peng Wang

pwang521@163.com

Supplementary Table S1. Reference genes for protein N-glycosylation reactions

---

>Och1 YGL038C
MSRKLSHLIATRKSKTIVVTVLLIYSLLTFHLSNKRLLSQFYPSKDDFKQTLLPTTSHSQDINLKKQITVNKKKNQLH
NLRDQLSFAFPYDSQAPIPQRVWQTWKVGADDKNFPSSFRTYQKTWSGSYSPDYQYSLISDDSIIPFLENLYAPV
PIVIQAFKLMPGNILKADFLRYLLLFARGGIYSDMDTMLLKPIDSWPSQNKSWLNNIIDLNKPIPYKNSKPSLLSS
DEISHQPGLVIGIEADPDRDDWSEWYARRIQFCQWTIQAKPGHPILRELILNITATTLASVQNPGVPVSEMIDPR
FEEDYNVNYRHKRRHDETYKHSELKNNKNVDGSDIMNWTGPGIFSDIIFEYMNNVLRYNSDILLINPNLNKND
EEGSESATTPAKDVDNDTLSKSTRKFYKKISESLQSSNSMPWEFFSFLKEPVIVDDVMVLPITSFSPDVGQMGAQ
SSDDKMAFVKHMFSGSWKEDADKNAGHK
>MNN9 YPL050C
MSLSLVSYRLRKNPWVNIFLPVLAIFLIYIIFFQRDQSLLGLNGQSISQHKWAHEKENTFYFPFTKKYKMPKYSYKK
KSGWLFNDHVEDIIPEGHIAHYDLNKLHSTSEAAVNKEHILILTPMQTFHQQYWDNLLQLNYPRELIELGFITPRT
ATGDLALKKLENAIKKVQTDKKTQRFSKITILRQNSQSFDKLMEKERHALDVQKERRAAMALARNELLFSTIGPH
TSWVLWLDADIIETPPSLIQDMTKHNKAILAANIYQRFYDEEKKQPSIRPYDFNNWQESDTGLEIASQMGDDEII
VEGYAEIATYRPLMAHFYDANGVPGEEMALDGVGGGCTLVKAEVHRDGAMFPNFPFYHLIETEGFAKMAKRL
NYDVFGLPNYLVYHIEEENH
>VAN1 YML115C
MGMFFNLRSNIKKKAMDNGLSLPISRNGSSNNIKDKRSEHNSNSLKGKYRYQPRSTPSKFQLTVSITSLIIIAVLSL
YLFISFLSGMGIGVSTQNGRSLLGSSKSSENYKTIDLEDEEYYDYDFEDIDPEVISKFDDGVQHYLISQFGSEVLTPK
DDEKYQRELNMLFDSTVEEYDLSNFEGAPNGLETRDHILLCIPLRNAADVLPLMFKHLMNLTYPHELIDLAFLVS
DCSEGDTTLDALIAYSRHLQNGTLSQIFQEIDAVIDSQTKGTDKLYLKYMDEGYINRVHQAFSPPFHENYDKPFRS
VQIFQKDFGQVIGQGFSDRHAVKVQGIRRKLMGRARNWLTANALKPYHSWVYWRDADVELCPGSVIQDLMS
KNYDVIVPNVWRPLPTFLGTEQPYDLNSWMESQEALALAKTLDEDDVIVEGYAEYPTWRVHLAYIRDAEGDPN
EAVDLDGVGGVSILAKAKIFRNGVQFPAFTFENHAETEAFGKMAKKMGYRVGGLPHYTIWHIYEPSDDDLKEIA
SREREKRRQSE
>ANP1 YEL036C
MKYNNRKLSFNPTTVSIAGTLLTVFFLTRLVLSFFSISLFQLVTFQGIFKPYVPDFKNTPSVEFYDLRNYQGNKDG
WQQGDRILFCVPLRDASEHLPMFFNHLNTMTYPHNLIDLSFLVSDSSDNTMGVLLSNLQMAQSQQDKSKRF
GNIEIYEKDFGQIIGQSFSDRHGFGAQGPRRKLMARARNWLGSVALKPYHSWVYWRDVDVETIPTTIMEDLM
HHDKDVIVPNVWRPLPDWLGNIQPYDLNSWKESEGGLQLADSLDEDAVIVEGYPEYATWRPHLAYMRDPNG
NPEDEMELDGIGGVSILAKAKVFRTGSHFPAFSFEKHAETEAFGRLSRRMNYNVIGLPHYVIWHIYEPSSDDLKH
MAWMAEEEKRKLEEERIREFYNKIWEIGFEDVRDQWNEERDSILKNIDSTLNNKVTVDWSEEGDGSELVDSKG
DFVSPNNQQQQQQQQQQQQQQQQQQQQQQLDGNPQGKPLDDNDKNKKKHPKEVPLDFDPDRN
>MNN10 YDR245W
MSSVPYNSQLPISNHLEYDEDEKKSRGSKLGLKYKMIYWRKTLCSSLARWRKLILLISLALFLFIWISDSTISRNPST
TSFQGQNSNDNKLSNTGSSINSKRYVPPYSKRSRWSFWNQDPRIVIILAANEGGGVLRWKNEQEWAIEGISIE
NKKAYAKRHGYALTIKDLTTSKRYSHEYREGWQKVDILRQTFREFPNAEWFWWLDLDTMIMEPSKSLEEHIFDR
LETLADRELKSFNPLNLRDDIPYVDYSEEMEFLITQDCGGFNLGSFLIKNSEWSKLLLDMWWDPVLYEQKHMV
WEHREQDALEALYENEPWIRSRIGFLPLRTINAFPPGACSEYSGDSRYFYSEKDHDFVVNMAGCNFGRDCWGE
MQYYTTLMEKLNRKWYTRFFFP
>MNN11 YJL183W
MAIKPRTKGKTYSSRSVGSQWFNRLGFKQNKYGTCKFLSIITAFVFILYFFSNRFYPISRSAGASYSPSHGLYINEIPA

SSRLIYPHVEHVPVLKQMTVRGLYITRLEVDGSKRLILKPEENALTDEEKKKTTDQILLVKHSFLDHGKLVYRKSND
APEVVVVTLIDFENYELETIIQIVQNRVDYAQKHQYGVYIRWIQEFLPVLENQNLAESYEFIKPLVIRAAMHAFPT
AKYIHFVDQDALLMNLDLSLQKYLLDPKIMDLALLKNVPVVANSNIKTYNHFEYSSAKIIIPHDADGNIDASSFVI
ANDFYGKALIDYLNDPLLRNFPWDNTGDKLSAAIGHILQWHPTLLGKTAIVIPKVLASQYDASLDQEGESGNGA
SNGDVYHYNEGDLAASFKGCRSRGTCASEIGHMYQKIKKS
>HOC1 YJR075W
MAKTTKRASSFRRLMIFAIIALISLAFGVRYLFHNSNATDLQKILQNLPKEISQSINSANNIQSSDSDLVQHFESLA
QEIRHQQEVQAKQFDKQRKILEKKIQDLKQTPPEATLRERIAMTFPYDSHVKFPAFIWQTWSNDEGPERVQDIK
GMWESKNPGFAHEVLNHDVINALVHHYFYSIPEILETYEALPSIILKIDFFKYLILLVHGGVYADIDTFPVQPIPNWI
PEELSPSDIGLIVGVEEDAQRADWRTKYIRRLQFGTWIIQAKPGHPVLREIISRIIETTLQRKRDDQLNVNLRNDL
NIMSWTGSGLWTDTIFTYFNDFMRSGVREKVTWKLFHNLNQPKLLSDVLVFPKFSFNCPNQIDNDDPHKKFYF
ITHLASQFWKNTPKVEQK
>MNN2 YBR015C
MLLTKRFSKLFKLTFIVLILCGLFVITNKYMDENTSVKEYKEYLDRYVQSYSNKYSSSSDAASADDSTPLRDNDEAG
NEKLKSFYNNVFNFLMVDSPKGSTAKQYNEACLLKGDIGDRPDHYKDLYKLSAKELSKCLELSPDEVASLTKSHKD
YVEHIATLVSPKGTYKGSGIATVGGGKFSLMAFLIIKTLRNMGTTLPVEVLIPPGDEGETEFCNKILPKYNSKCIYVS
DILPRETIEKFVFKGYQFKSLALIASSFENLLLLDADNFPIKPLDNIFNEEPYVSTGLVMWPDFWRRTTHPLYYDIA
GIAVDKKKRVRNSRDDITPPAVYTKDLKDLSDVPLSDLDGTIPDVSTESGQLMINKTKHLATALLSLFYNVNGPT
WYYPIFSQKAAGEGDKETFIAAANFYGLSFYQVRTRTGVEGYHDEDGFHGVAMLQHDFVQDYGRYLNAMESI
GNKYGGTKSADAIKFDKNYSLEKYTEEFFDNEDLNAKNHVDVMFIHSNFPKFDPYDLSKSNFLTTNGKPARSYTA
LKKVKNYDIELENFKVLNEYVCVNKNPFKYLDDLLGQDKTEWKRVCGYITDRLAFLESTHDKAIAGK
>MNN5 YJL186W
MLIRLKKRKILQVIVSAVVLILFFCSVHNDVSSSWLYGKKLRLPVLTRSNLKNNFYTTLVQAIVENKPADSSPDLSKL
HGAEGCSFANNVAAHDSGHDSDLSYESLSKCYNLNKTVQESLREVHSKFTDTLSGKLNFSIPQREALFSGSEGIV
TIGGGKYSVLAYTMIKKLRDTGTTLPIEVIIPPQDEGEDDFCKNWLPKFNGKCIYFSDIVPSKPLSDLKLTHFQLKVF
GLIISSFKRIIFLDADNYAVKNLDLAFNTTSFNDTGLILWPDFWRRVTPPAFYNIIGSSINIGKRVRFVSDDISPVSRY
DPFVSNSNDYTPKERQEHFLKHVPLHDLDGTMPDLSSESGQMVIDKIRHFNTLLLALYYNVYGPTWYYKMISQ
GTAGEGDKDTFVAAAHALNMPYYQVRTNFEFDGFFYQKDDYKGLALLQHDFEQDYKQYQKAQQKVKANIEEF
SKLDPDYTLDNGFLKTLMVNDDGSDLDIMFIHASFYKADPWTLYHENRFIGPNGEQVRGFRKPHRYGMDFELF
LFNDMRGSFCTTPKSQVIKFKYFTDKVNTPEWDAMCEYLTNHVNYLESTHKEAMGEKN
>MNN1 YER001W
MLALRRFILNQRSLRSCTIPILVGALIIILVLFQLVTHRNDALIRSSNVNSTNKKTLKDADPKVLIEAFGSPEVDPVDT
IPVSPLELVPFYDQSIDTKRSSSWLINKKGYYKHFNELSLTDRCKFYFRTLYTLDDEWTNSVKKLEYSINDNEGVDE
GKDANGNPMDEKSERLYRRKYDMFQAFERIRAYDRCFMQANPVNIQEIFPKSDKMSKERVQSKLIKTLNATFP
NYDPDNFKKYDQFEFEHKMFPFINNFTTETFHEMVPKITSPFGKVLEQGFLPKFDHKTGKVQEYFKYEYDPSKT
FWANWRDMSAKVAGRGIVLSLGSNQFPLAVKFIASLRFEGNTLPIQVVYRGDELSQELVDKLIYAARSPDFKPVE
NNYDNSTNVPQEIWFLDVSNTIHPKWRGDFGSYKSKWLVVLLNLLQEFVFLDIDAISYEKIDNYFKTTEYQKTGT
VFYRERALRENVNERCIARYETLLPRNLESKNFQNSLLIDPDHALNECDNTLTTEEYIFKAFFHHRRQHQLEAGLF
AVDKSKHTIPLVLAAMIHLAKNTAHCTHGDKENFWLGFLAAGHTYALQGVYSGAIGDYVKKTDLNGKRQEAAV
EICSGQIAHMSTDKKTLLWVNGGGTFCKHDNAAKDDWKKDGDFKKFKDQFKTFEEMEKYYYITPISSKYVILP
DPKSDDWHRASAGACGGYIWCATHKTLLKPYSYNHRTTHGELITLDEEQRLHIDAVNTVWSHANKDNTRSFTE
EEIKELENSRHEQS
>MNN6 YPL053C
MHVLLSKKIARFLLISFVFVLALMVTINHPKTKQMSEQYVTPYLPKSLQPIAKISAEEQRRIQSEQEEAELKQSLEG

EAIRNATVNAIKEKIKSYGGNETTLGFMVPSYINHRGSPPKACFVSLITERDSMTQILQSIDEVQVKFNKNFAYPW
VFISQGELDGMKQEMIRQAITDSMNGDPELINIKFAEIPADEWVYPEWIDENKAAESLISLANVPDGDSRAVRY
QARYFAGFFWRHPVLDEFDWYWRVDPGIKLYCDIDHDLFRWMQDEGKVFGFTLSMSEAKEANEKIWDVTKK
FAKDFPKFISENNFKSFITKKDSEDFNNCEFTSNFEIGNLNFYRSPAYRKFFNYIDEEGGIFYWKWSDSIIHTIGLS
MLLPKDKIHFFENIGFHYDKYNNCPLNDDIWNQYNCNCDQGNDFTFRSGSCGGHYFDIMKKDKPEGWDRLP
>MNS1 AT1G51590.1
MARSRSISGYGIWKYLNPAYYLRRPRRLALLFIVFVSVSMLVWDRINLAREHEVEVFKLNEEVSRLEQMLEELNG
GVGNKPLKTLKDAPEDPVDKQRRQKVKEAMIHAWSSYEKYAWGKDELQPRTKDGTDSFGGLGATMVDSLDT
LYIMGLDEQFQKAREWVASSLDFDKDYDASMFETTIRVVGGLLSAYDLSGDKMFLEKAKDIADRLLPAWNTPT
GIPYNIINLRNGNAHNPSWAAGGDSILADSGTEQLEFIALSQRTGDPKYQQKVEKVITELNKNFPADGLLPIYINP
DNANPSYSTTTFGAMGDSFYEYLLKVWVQGNKTSAVKPYRDMWEKSMKGLLSLVKKSTPSSFTYICEKNGNN
LIDKMDELACFAPGMLALGASGYGPDEEKKFLSLAGELAWTCYNFYQSTPTKLAGENYFFTAGQDMSVGTSW
NILRPETVESLFYLWRLTGNKTYQEWGWNIFQAFEKNSRVESGYVGLKDVNTGAKDNKMQSFFLAETLKYLYLL
FSPSSVISLDEWVFNTEAHPLKIVARNDPRKPTIALRQRKFGHQINV
>GnTI AT4G38240.1
MARISCDLRFLLIPAAFMFIYIQMRLFQTQSQYADRLSSAIESENHCTSQMRGLIDEVSIKQSRIVALEDMKNRQ
DEELVQLKDLIQTFEKKGIAKLTQGGQMPVAAVVVMACSRADYLERTVKSVLTYQTPVASKYPLFISQDGSDQA
VKSKSLSYNQLTYMQHLDFEPVVTERPGELTAYYKIARHYKWALDQLFYKHKFSRVIILEDDMEIAPDFFDYFEAA
ASLMDRDKTIMAASSWNDNGQKQFVHDPYALYRSDFFPGLGWMLKRSTWDELSPKWPKAYWDDWLRLKE
NHKGRQFIRPEVCRTYNFGEHGSSLGQFFSQYLEPIKLNDVTVDWKAKDLGYLTEGNYTKYFSGLVRQARPIQG
SDLVLKAQNIKDDVRIRYKDQVEFERIAGEFGIFEEWKDGVPRTAYKGVVVFRIQTTRRVFLVGPDSVMQLGIRN
S
>GMII AT5G14950.1
MPFSSYIGNSRRSSTGGGTGGWGQSLLPTALSKSKLAINRKPRKRTLVVNFIFANFFVIALTVSLLFFLLTLFHFGVP
GPISSRFLTSRSNRIVKPRKNINRRPLNDSNSGAVVDITTKDLYDRIEFLDTDGGPWKQGWRVTYKDDEWEKEK
LKIFVVPHSHNDPGWKLTVEEYYQRQSRHILDTIVETLSKDSRRKFIWEEMSYLERWWRDASPNKQEALTKLVK
DGQLEIVGGGWVMNDEANSHYFAIIEQIAEGNMWLNDTIGVIPKNSWAIDPFGYSSTMAYLLRRMGFENMLI
QRTHYELKKDLAQHKNLEYIWRQSWDAMETTDIFVHMMPFYSYDIPHTCGPEPAICCQFDFARMRGFKYELC
PWGKHPVETTLENVQERALKLLDQYRKKSTLYRTNTLLIPLGDDFRYISIDEAEAQFRNYQMLFDHINSNPSLNA
EAKFGTLEDYFRTVREEADRVNYSRPGEVGSGQVVGFPSLSGDFFTYADRQQDYWSGYYVSRPFFKAVDRVLE
HTLRGAEIMMSFLLGYCHRIQCEKFPTSFTYKLTAARRNLALFQHHDGVTGTAKDYVVQDYGTRMHTSLQDLQI
FMSKAIEVLLGIRHEKEKSDQSPSFFEAEQMRSKYDARPVHKPIAAREGNSHTVILFNPSEQTREEVVTVVVNRA
EISVLDSNWTCVPSQISPEVQHDDTKLFTGRHRLYWKASIPALGLRTYFIANGNVECEKATPSKLKYASEFDPFPC
PPPYSCSKLDNDVTEIRNEHQTLVFDVKNGSLRKIVHRNGSETVVGEEIGMYSSPESGAYLFKPDGEAQPIVQPD
GHVVTSEGLLVQEVFSYPKTKWEKSPLSQKTRLYTGGNTLQDQVVEIEYHVELLGNDFDDRELIVRYKTDVDNK
KVFYSDLNGFQMSRRETYDKIPLQGNYYPMPSLAFIQGSNGQRFSVHSRQSLGVASLKEGWLEIMLDRRLVRD
DGRGLGQGVMDNRAMTVVFHLLAESNISQADPASNTNPRNPSLLSHLIGAHLNYPINTFIAKKPQDISVRVPQ
YGSFAPLAKPLPCDLHIVNFKVPRPSKYSQQLEEDKPRFALILNRRAWDSAYCHKGRQVNCTSMANEPVNFSD
MFKDLAASKVKPTSLNLLQEDMEILGYDDQELPRDSSQPREGRVSISPMEIRAYKLELRPHK
>GnTII AT2G05320.1
MANLWKKQRLRDTGLCRLGILFAVTLSIVLMLVSVPRTALNGSSIDDDLDGLDKDLEAKLNASLLSVARGNRMSL
RLHRRNHFSPRNTDLFPDLAKDRVVIVLYVHNRAQYFRVTVESLSKVKGISETLLIVSHDGYFEEMNRIVESIKFC
QVKQIFSPYSPHIYRTSFPGVTLNDCKNKGDEAKGHCEGNPDQYGNHRSPKIVSLKHHWWWMMNTVWDGL
EETKGHEGHILFIEEDHFLFPNAYRNIQTLTRLKPAKCPDCFAANLAPSDVKSRGEGLESLVAERMGNVGYSFNRS

VWENIHQKAREFCFFDDYNWDITMWATVFPSFGSPVYTLRGPRTSAVHFGKCGLHQGRGDEGDCIDNGVVN
IEVKETDKVVNIKEGWGVRVYKHQAGYKAGFEGWGGWGDDRDRHLCLDFATMYRYSSSSASP
>beta1,2-XylT AT5G55500.1
MSKRNPKILKIFLYMLLLNSLFLIIYFVFHSSSFSPEQSQPPHIYHVSVNNQSAIQKPWPILPSYLPWTPPQRNLPT
GSCEGYFGNGFTKRVDFLKPRIGGGGEGSWFRCFYSETLQSSICEGRNLRMVPDRIVMSRGGEKLEEVMGRKE
EEELPAFRQGAFEVAEEVSSRLGFKRHRRFGGGEGGSAVSRRLVNDEMLNEYMQEGGIDRHTMRDLVASIRAV
DTNDFVCEEWVEEPTLLVTRFEYANLFHTVTDWYSAYVSSRVTGLPNRPHVVFVDGHCTTQLEETWTALFSGIR
YAKNFTKPVCFRHAILSPLGYETALFKGLSGEIDCKGDSAHNLWQNPDDKRTARISEFGEMIRAAFGLPVNRHRS
LEKPLSSSSSSASVYNVLFVRREDYLAHPRHGGKVQSRLINEEEVFDSLHHWVATGSTGLTKCGINLVNGLLAHM
SMKDQVRAIQDASVIIGAHGAGLTHIVSATPNTTIFEIISVEFQRPHFELIAKWKGLEYHAMHLANSRAEPTAVIE
KLTEIMKSLGC
>alpha1,3-FucT_AT3G19280.1
MGVFSNLRGPKIGLTHEELPVVANGSTSSSSSPSSFKRKVSTFLPICVALVVIIEIGFLCRLDNASLVDTLTHFFTKSS
SDLKVGSGIEKCQEWLERVDSVTYSRDFTKDPIFISGSNKDFKSCSVDCVMGFTSDKKPDAAFGLSHQPGTLSIIR
SMESAQYYQENNLAQARRKGYDIVMTTSLSSDVPVGYFSWAEYDIMAPVQPKTEKALAAAFISNCAARNFRLQ
ALEALMKTNVKIDSYGGCHRNRDGSVEKVEALKHYKFSLAFENTNEEDYVTEKFFQSLVAGSVPVVVGAPNIEE
FAPSPDSFLHIKQMDDVKAVAKKMKYLADNPDAYNQTLRWKHEGPSDSFKALIDMAAVHSSCRLCIFVATRIRE
QEEKSPEFKRRPCKCTRGSETVYHLYVRERGRFDMESIFLKDGNLTLEALESAVLAKFMSLRYEPIWKKERPASLR
GDGKLRVHGIYPIGLTQRQALYNFKFEGNSSLSTHIQRNPCPKFEVVFV
>beta1,3-GalT AT1G26810.1
MKRFYGGLLVVSMCMFLTVYRYVDLNTPVEKPYITAAASVVVTPNTTLPMEWLRITLPDFMKEARNTQEAISG
DDIAVVSGLFVEQNVSKEEREPLLTWNRLESLVDNAQSLVNGVDAIKEAGIVWESLVSAVEAKKLVDVNENQTR
KGKEELCPQFLSKMNATEADGSSLKLQIPCGLTQGSSITVIGIPDGLVGSFRIDLTGQPLPGEPDPPIIVHYNVRLL
GDKSTEDPVIVQNSWTASQDWGAEERCPKFDPDMNKKVDDLDECNKMVGGEINRTSSTSLQSNTSRGVPVA
REASKHEKYFPFKQGFLSVATLRVGTEGMQMTVDGKHITSFAFRDTLEPWLVSEIRITGDFRLISILASGLPTSEES
EHVVDLEALKSPTLSPLRPLDLVIGVFSTANNFKRRMAVRRTWMQYDDVRSGRVAVRFFVGLHKSPLVNLELW
NEARTYGDVQLMPFVDYYSLISWKTLAICIFGTEVDSAKFIMKTDDDAFVRVDEVLLSLSMTNNTRGLIYGLINS
DSQPIRNPDSKWYISYEEWPEEKYPPWAHGPGYIVSRDIAESVGKLFKEGNLKMFKLEDVAMGIWIAELTKHGL
EPHYENDGRIISDGCKDGYVVAHYQSPAEMTCLWRKYQETKRSLCCREW
>alpha1,4-FucT AT1G71990.1
MPMRYLNAMAALLMMFFTLLILSFTGILEFPSASTSMEHSIDPEPKLSDSTSDPFSDVLVAYKKWDFEVGCARFR
ENHKDAILGNVSSGSLQEFGCGKLKMKHVKVLVKGWTWIPDNLENLYSCRCGMTCLWTKSSVLADSPDALLFE
TTTPPLQRRVGDPLRVYMELEAGRKRSGREDIFISYHAKDDVQTTYAGSLFHNNRNYHISPHKNNDVLVYWSSS
RCLPHRDRLAKSLLDLIPHHSFGKCLNNVGGLDSALSMYPECVAEHNAEAKWYDHLHCAMSHYKFVLAIENTA
VESYVTEKLFYALDSGSVPIYFGASNVQDFVPPHSVIDGSKFGSMQELAAYVKRLGDDPVAYSEYHAWRRCGL
MGNYGKTRAVSLDTLPCRLCEEISRRGGKNAGV
>beta1,4-GALT ENSG00000086062
MRLREPLLSGSAAMPGASLQRACRLLVAVCALHLGVTLVYYLAGRDLSRLPQLVGVSTPLQGGSNSAAAIGQSS
GELRTGGARPPPPLGASSQPRPGGDSSPVVDSGPGPASNLTSVPVPHTTALSLPACPEESPLLVGPMLIEFNMP
VDLELVAKQNPNVKMGGRYAPRDCVSPHKVAIIIPFRNRQEHLKYWLYYLHPVLQRQQLDYGIYVINQAGDTIF
NRAKLLNVGFQEALKDYDYTCFVFSDVDLIPMNDHNAYRCFSQPRHISVAMDKFGFRLVFRGMSISRPNAVVG
RCRMIRHSRDKKNEPNPQRFDRIAHTKETMLSDGLNSLTYQVLDVQRYPLYTQITVDIGTPS
>alpha2,6-SialT ENSG00000117069
MKTLMRHGLAVCLALTTMCTSLLLVYSSLGGQKERPPQQQQQQQQQQQQASATGSSQPAAESSTQQRPGVP

AGPRPLDGYLGVADHKPLKMHCRDCALVTSSGHLLHSRQGSQIDQTECVIRMNDAPTRGYGRDVGNRTSLRVI
AHSSIQRILRNRHDLLNVSQGTVFIFWGPSSYMRRDGKGQVYNNLHLLSQVLPRLKAFMITRHKMLQFDELFK
QETGKDRKISNTWLSTGWFTMTIALELCDRINVYGMVPPDFCRDPNHPSVPYHYYEPFGPDECTMYLSHERG
RKGSHHRFITEKRVFKNWARTFNIHFFQPDWKPESLAINHPENKPVF

>GnTIII ENSG00000128268

MRRYKLFLMFCMAGLCLISFLHFFKTLSYVTFPRELASLSPNLVSSFFWNNAPVTPQASPEPGGPDLLRTPLYSHS
PLLQPLPPSKAAEELHRVDLVLPEDTTEYFVRTKAGGVCFKPGTKMLERPPPGRPEEKPEGANGSSARRPPRYLL
SARERTGGRGARRKWVECVCLPGWHGPSCGVPTVVQYSNLPTKERLVPREVPRRVINAINVNHEFDLLDVRFH
ELGDVVDAFVVCESNFTAYGEPRPLKFREMLTNGTFEYIRHKVLYVFLDHFPPGGRQDGWIADDYLRTFLTQDG
VSRLRNLRPDDVFIIDDADEIPARDGVLFLKLYDGWTEPFAFHMRKSLYGFFWKQPGTLEVVSGCTVDMLQAVY
GLDGIRLRRRQYYTMPNFRQYENRTGHILVQWSLGSPLHFAGWHCSWCFTPEGIYFKLVSAQNGDFPRWGDY
EDKRDLNYIRGLIRTGGWFDGTQQEYPPADPSEHMYAPKYLLKNYDRFHYLLDNPYQEPRSTAAGGWRHRGP
EGRPPARGKLDEAEV

>GnTIV ENSG00000071073

MNYSITVSIVMGIPTVKREVKSYLIETLHSLIDNLYPEEKLDCVIVVFIGETDIDYVHGVVANLEKEFSKEISSGLVEV
ISPPESYYPDLTNLKETFGDSKERVRWRTKQNLDYCFLMMYAQEKGIYYIQLEDDIIVKQNYFNTIKNFALQLSSEE
WMILEFSQLGFIGKMFQAPDLTLIVEFIFMFYKEKPIDWLLDHILWVKVCNPEKDAKHCDRQKANLRIRFRPSLF
QHVGLHSSLSGKIQKLTDKDYMKPLLLKIHVNPPAEVSTSLKVYQGHTLEKTYMGEDFFWAITPIAGDYILFKFDK
PVNVESYLFHSGNQEHPGDILLNTTVEVLPFKSEGLEISKETKDKRLEDGYFRIGKFENGVAEGMVDPSLNPISAF
RLSVIQNSAVWAILNEQRVFSGWWQKSRDKAEGPQAPLLF

>GnTV ENSG00000152127

MALFTPWKLSSQKLGFFLVTFGFIWGMMLLHFTIQQRTQPESSSMLREQILDLSKRYIKALAEENRNVVDGPYA
GVMTAYDLKKTLAVLLDNILQRIGKLESKVDNLVVNGTGTNSTNSTTAVPSLVALEKINVADIINGAQEKCVLPPM
DGYPHCEGKIKWMKDMWRSDPCYADYGVDGSTCSFFIYLSEVENWCPHLPWRAKNPYEEADHNSLAEIRTD
FNILYSMMKKHEEFRWMRLRIRRMADAWIQAIKSLAEKQNLEKRKRKKVLVHLGLLTKESGFKIAETAFSGGPL
GELVQWSDLITSLYLLGHDIRISASLAELKEIMKKVVGNRSGCPTVGDRIVELIYIDIVGLAQFKKTLGPSWVHYQC
MLRVLDSFGTEPEFNHANYAQSKGHKTPWGKWNLNPQQFYTMFPHTPDNSFLGFVVEQHLNSSDIHHINEIK
RQNQSLVYGKVDSFWKNKKIYLDIIHTYMEVHATVYGSSTKNIPSYVKNHGILSGRDLQFLLRETKLFVGLGFPYE
GPAPLEAIANGCAFLNPKFNPPKSSKNTDFFIGKPTLRELTSQHPYAEVFIGRPHVWTVDLNNQEEVEDAVKAIL
NQKIEPYMPYEFTCEGMLQRINAFIEKQDFCHGQVMWPPLSALQVKLAEPGQSCKQVCQESQLICEPSFFQHL
NKDKDMLKYKVTCQSSELAKDILVPSFDPKNKHCVFQGDLLLFSCAGAHPRHQRVCPCRDFIKGQVALCKDCL

Supplementary Table S2. Species included in the study for gene identification invloved in protein N-glycosylation.

Fungi

24 species. All have chromosome-level genome assembly data available.

abbr.	species	data source
aspni	*Aspergillus nidulans FGSC A4*	AspGD (http://aspergillusgenome.org/)
botci	*Botrytis cinerea B05.10*	Ensembl
cryne	*Cryptococcus neoformans var. grubii H99*	https://www.broadinstitute.org
debha	*Debaryomyces hansenii CBS767*	JGI
enccu	*Encephalitozoon cuniculi GB-M1*	JGI
erego	*Eremothecium gossypii ATCC 10895*	Ensembl
fusox	*Fusarium oxysporum sp. lycopersici 4287*	JGI
kazaf	*Kazachstania africana CBS 2517*	JGI
klula	*Kluyveromyces lactis*	JGI
lacth	*Lachancea thermotolerans CBS 6340*	Ensembl
magor	*Magnaporthe oryzae 70-15*	JGI
milfa	*Millerozyma farinosa*	Ensembl
mycth	*Myceliophthora thermophila ATCC 42464*	Ensembl
nauca	*Naumovozyma castellii CBS 4309*	JGI
neucr	*Neurospora crassa OR74A*	Ensembl
ogapa	*Ogataea parapolymorpha DL-1*	JGI
pench	*Penicillium chrysogenum*	JGI
sacce	*Saccharomyces cerevisiae S288c*	yeastgenome.org
schpo	*Schizosaccharomyces pombe 972h-*	PomBase
spore	*Sporisorium reilianum SRZ2*	JGI
tetph	*Tetrapisispora phaffii CBS 4417*	Ensembl
thite	*Thielavia terrestris NRRL 8126*	Ensembl
torde	*Torulaspora delbrueckii*	JGI
ustma	*Ustilago maydis 521*	Ensembl
verda	*Verticillium dahliae JR2*	Ensembl
yarli	*Yarrowia lipolytica (strain CLIB122)*	http://www.genolevures.org/
zygro	*Zygosaccharomyces rouxii*	http://www.genolevures.org/
zymtr	*Zymoseptoria tritici IPO323*	Ensembl

Animals

34 species. All have chromosome-level genome assembly data available.

abbr.	species	data source
anoca	*Anolis carolinensis*	Ensembl
anoga	*Anopheles gambiae*	Ensembl

apime	*Apis mellifera*	Ensembl
bosta	*Bos taurus*	Ensembl
caeel	*Caenorhabditis elegans*	Ensembl
calja	*Callithrix jacchus*	Ensembl
chlsa	*Chlorocebus sabaeus*	Ensembl
cioin	*Ciona intestinalis*	Ensembl
danre	*Danio rerio*	Ensembl
drome	*Drosophila melanogaster*	Ensembl
equca	*Equus caballus*	Ensembl
felca	*Felis catus*	Ensembl
fical	*Ficedula albicollis*	Ensembl
galga	*Gallus gallus*	Ensembl
gorgo	*Gorilla gorilla*	Ensembl
homsa	*Homo sapiens*	Ensembl
lepoc	*Lepisosteus oculatus*	Ensembl
macmu	*Macaca mulatta*	Ensembl
melga	*Meleagris gallopavo*	Ensembl
mondo	*Monodelphis domestica*	Ensembl
musmu	*Mus musculus*	Ensembl
nasvi	*Nasonia vitripennis*	Ensembl
oreni	*Oreochromis niloticus*	Ensembl
ornan	*Ornithorhynchus anatinus*	Ensembl
orycu	*Oryctolagus cuniculus*	Ensembl
oryla	*Oryzias latipes*	Ensembl
oviar	*Ovis aries*	Ensembl
pantr	*Pan troglodytes*	Ensembl
papan	*Papio anubis*	Ensembl
ponab	*Pongo abelii*	Ensembl
ratno	*Rattus norvegicus*	Ensembl
sussc	*Sus scrofa*	Ensembl
taegu	*Taeniopygia guttata*	Ensembl
takru	*Takifugu rubripes*	Ensembl

Plants

28 species. All have chromosome-level genome assembly data available, except chlre, volca, klefl, phypa, selmo, picab and ambtr.

abbr.	species	data source
anaco	*Ananas comosus*	Phytozome
arath	*Arabidopsis thaliana*	TAIR
betvu	*Beta vulgaris subsp. vulgaris*	Phytozome
bradi	*Brachypodium distachyon*	Phytozome
capan	*Capsicum annuum*	Solgenomics
cicar	*Cicer arietinum*	Phytozome
citsi	*Citrus sinensis*	Phytozome

elagu	*Elaeis guineensis*	Phytozome
eutsa	*Eutrema salsugineum*	Phytozome
frave	*Fragaria vesca subsp. vesca*	Phytozome
gosra	*Gossypium raimondii*	Phytozome
leepe	*Leersia perrieri*	Phytozome
maldo	*Malus domestica*	Phytozome
medtr	*Medicago truncatula*	Phytozome
orysa	*Oryza sativa Japonica Group*	http://rice.plantbiology.msu.edu
phavu	*Phaseolus vulgaris*	Phytozome
prumu	*Prunus mume*	Phytozome
solly	*Solanum lycopersicum*	Solgenomics
sorbi	*Sorghum bicolor*	Phytozome
theca	*Theobroma cacao*	Phytozome
vitvi	*Vitis vinifera*	Phytozome
chlre	*Chlamydomonas reinhardtii*	Phytozome
volca	*Volvox carteri*	Phytozome
klefl	*Klebsormidium flaccidum*	Phytozome
phypa	*Physcomitrella pattens*	Phytozome
selmo	*Selaginella moellendorffii*	Phytozome
picab	*Picea abies*	Phytozome
ambtr	*Amborella trichopoda*	http://www.amborella.org/

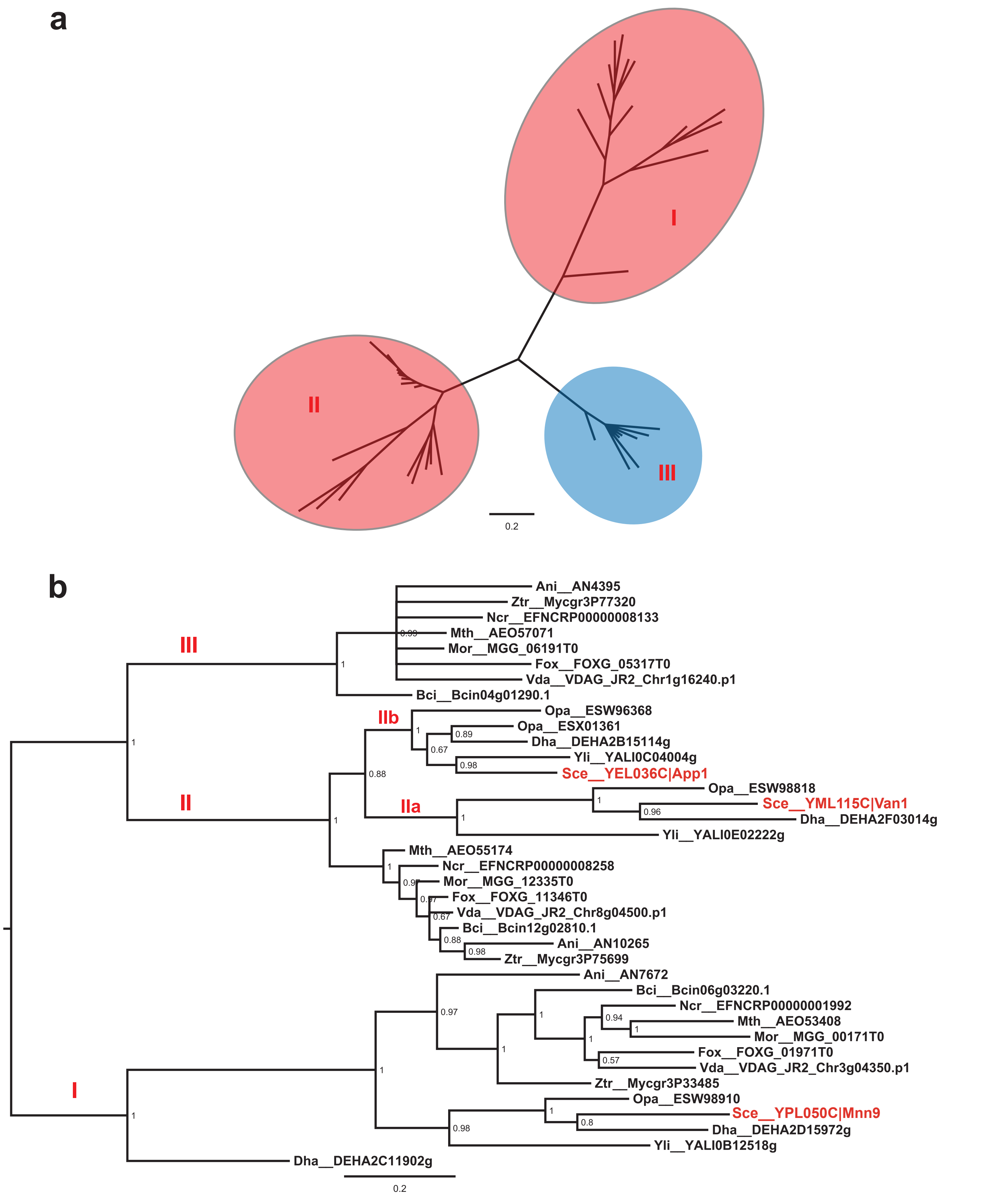

Supplementary Figure S1. Phylogeny of the genes containing domain PF03452, which include Man Pol-I (in Clade IIa) and Man Pol-II (in Clade IIb). a.Unrooted phylogenetic tree of the genes without tip labels. Clade I and II, where known genes Mnn9, Van1 and App1 reside, are shaded in red, while Clade III is shaded in blue. b. Phylogenetic tree with gene locus IDs labeled in tips and Bayesian posterior probabilities labeled at nodes. Clade labels are in red, and also genes with known functions are labeled in red, with common names appending locus IDs separated by vertical lines.

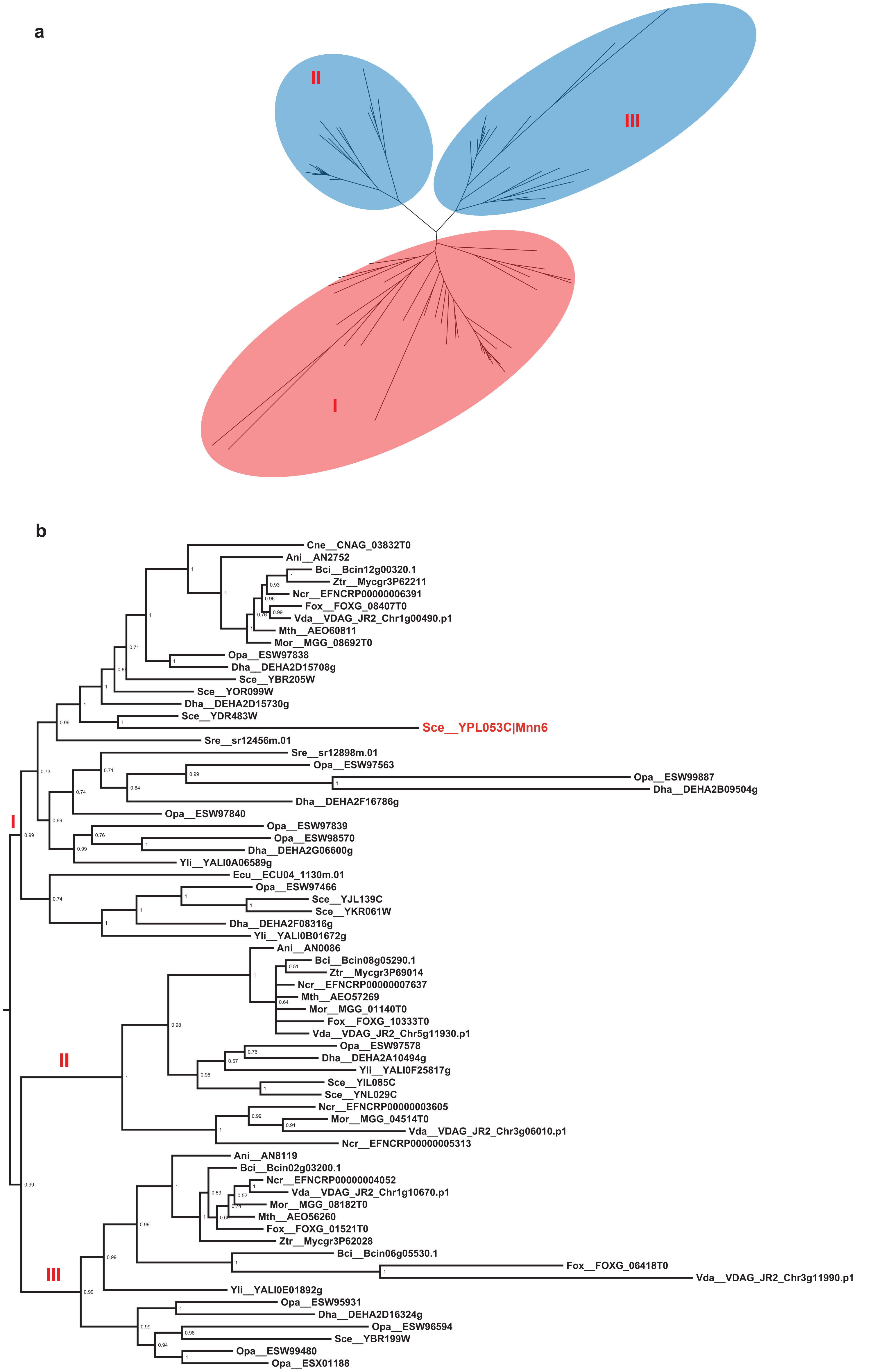

Supplementary Figure S2. Phylogeny of the genes containing domain PF01793, which include Mnn6 (in Clade I). a.Unrooted phylogenetic tree of the genes without tip labels. Clade I, where known gene Mnn6 residues, are shaded in red, while Clades II and III are shaded in blue. b. Phylogenetic tree with gene locus IDs labeled in tips and Bayesian posterior probabilities labeled at nodes. Clade labels are in red, and YPL053C, the known gene, is labeled in red too, with common names appending locus IDs separated by vertical lines.

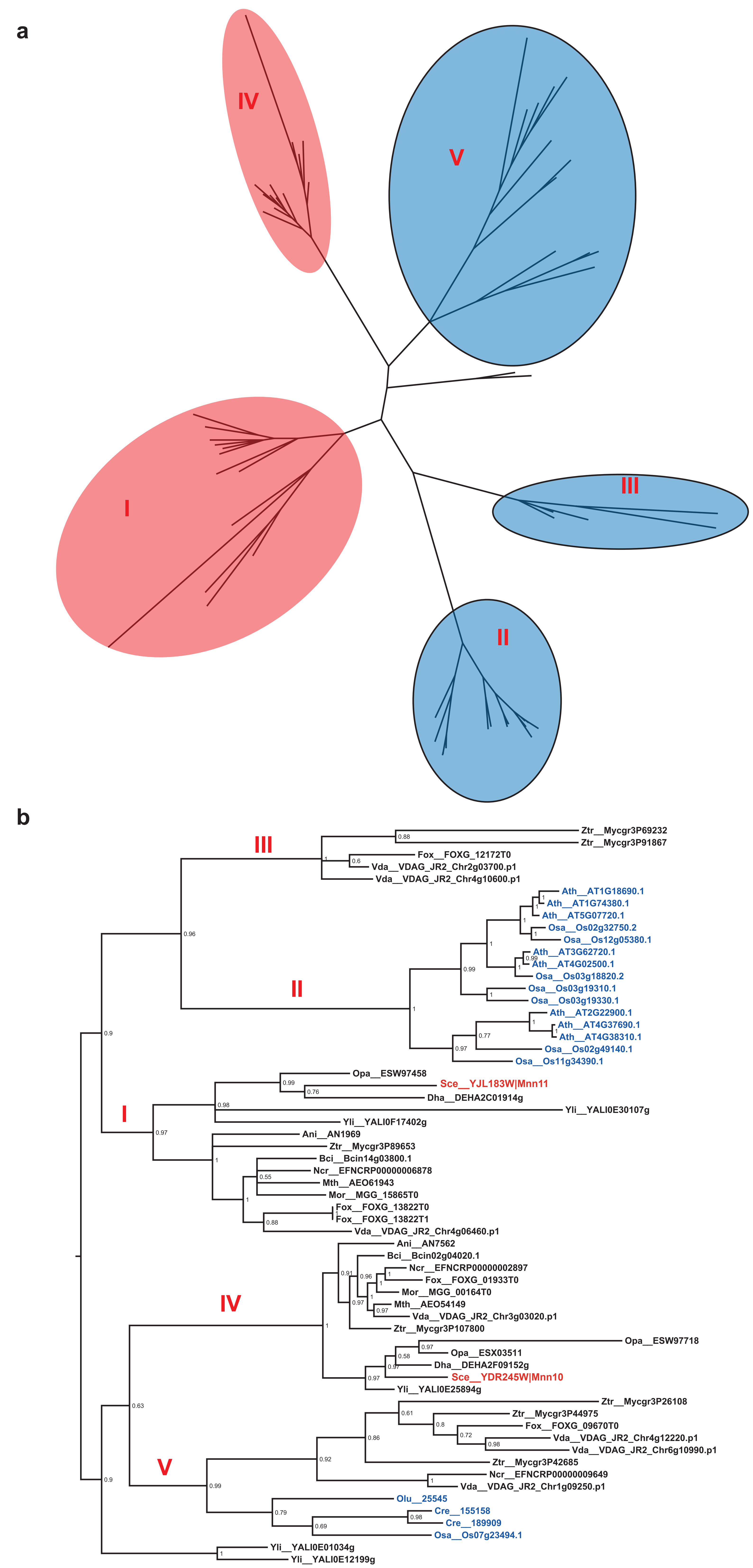

Supplementary Figure S3. Phylogeny of the genes containing domain PF05637, which include Mnn10 (in Clade I) and Mnn 11 (in Clade IV). a.Unrooted phylogenetic tree of the genes without tip labels. Clades I and IV, where known genes Mnn11 and Mnn10 reside, respectively, are shaded in red, while other clades are shaded in blue. b. Phylogenetic tree with gene locus IDs labeled in tips and Bayesian posterior probabilities labeled at nodes. Clade labels are in red, and also the two genes with known functions are labeled in red, with common names appending locus IDs separated by vertical lines. Tip labels of plant genes are in blue.

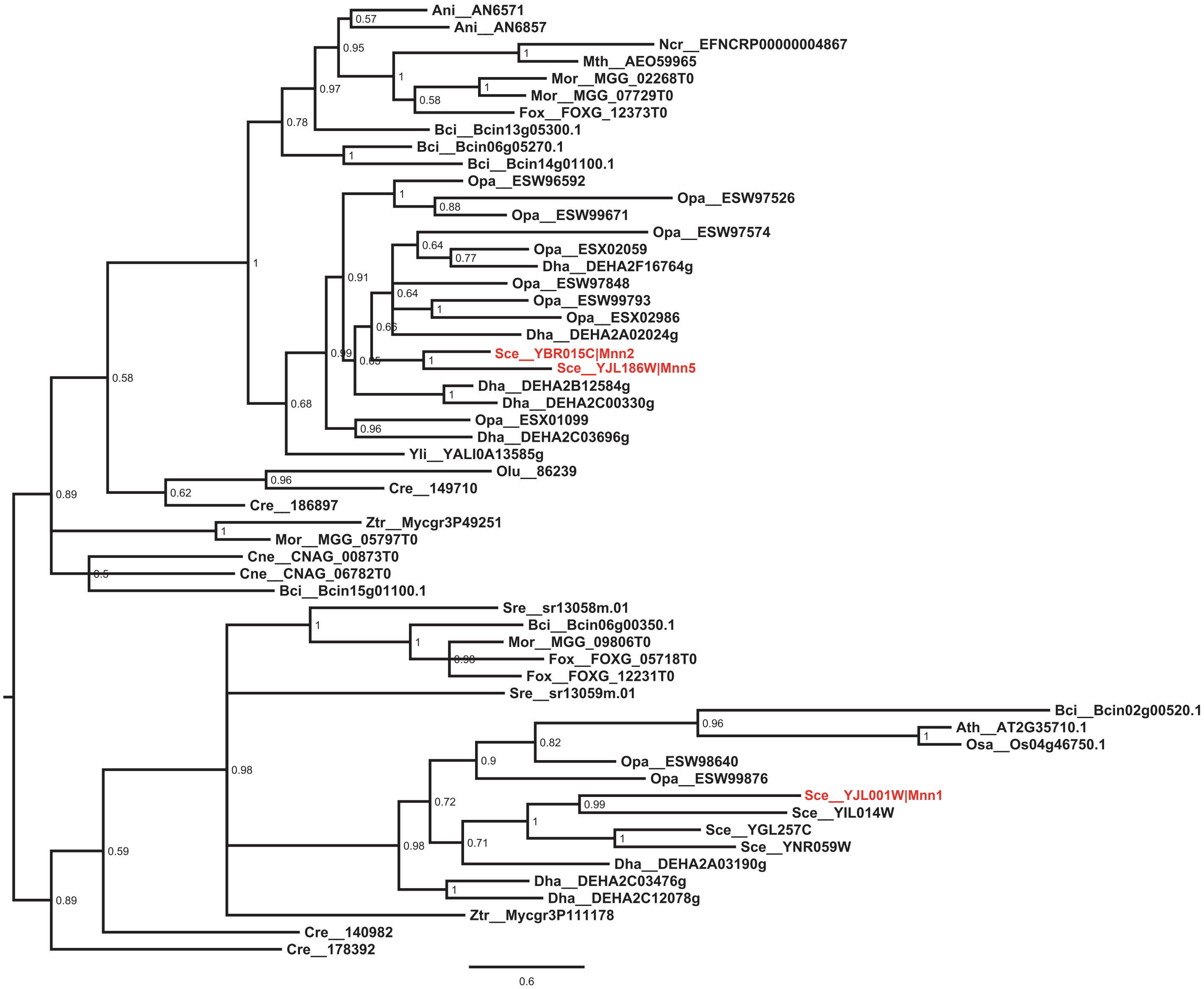

Supplementary Figure S4. Phylogeny of the genes containing domain PF11051, in which yeast mannosyltransferase genes are included. Known yeast mannosyltransferase genes, Mnn1, Mnn2 and Mnn5 are labeled in red.

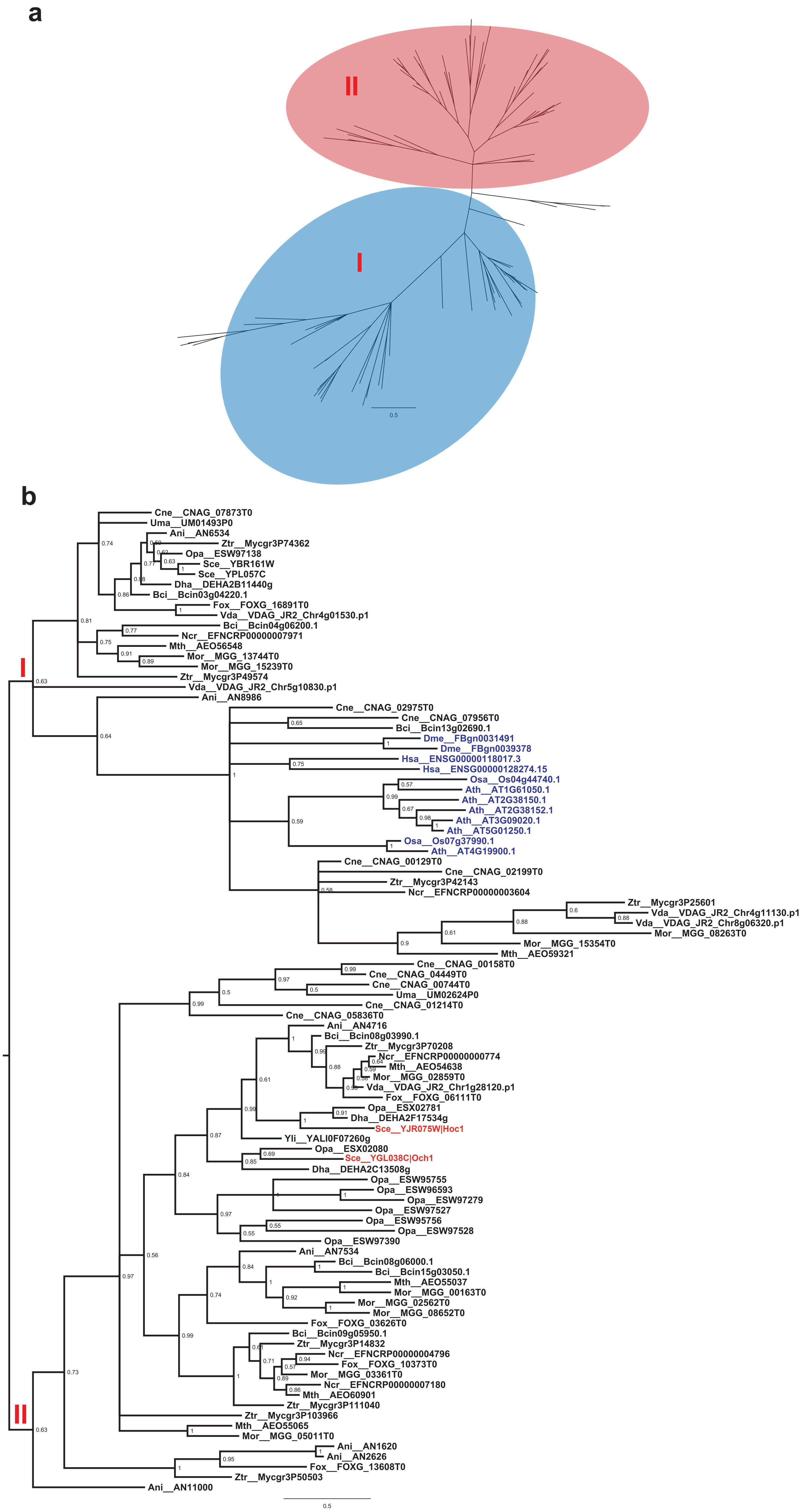

Supplementary Figure S5. Phylogeny of the genes containing domain PF01793, which include Och1 and Hoc1 (both in Clade II, as shaed in red). a.Unrooted phylogenetic tree of the genes without tip labels. b. Phylogenetic tree with gene locus IDs labeled in tips and Bayesian posterior probabilities labeled at nodes. Clade labels are in red, and also Hoc1 and Och1, the genes with known functions, are labeled in red with common names appending locus IDs separated by vertical lines. Tip labels of plant genes are labeled in blue.

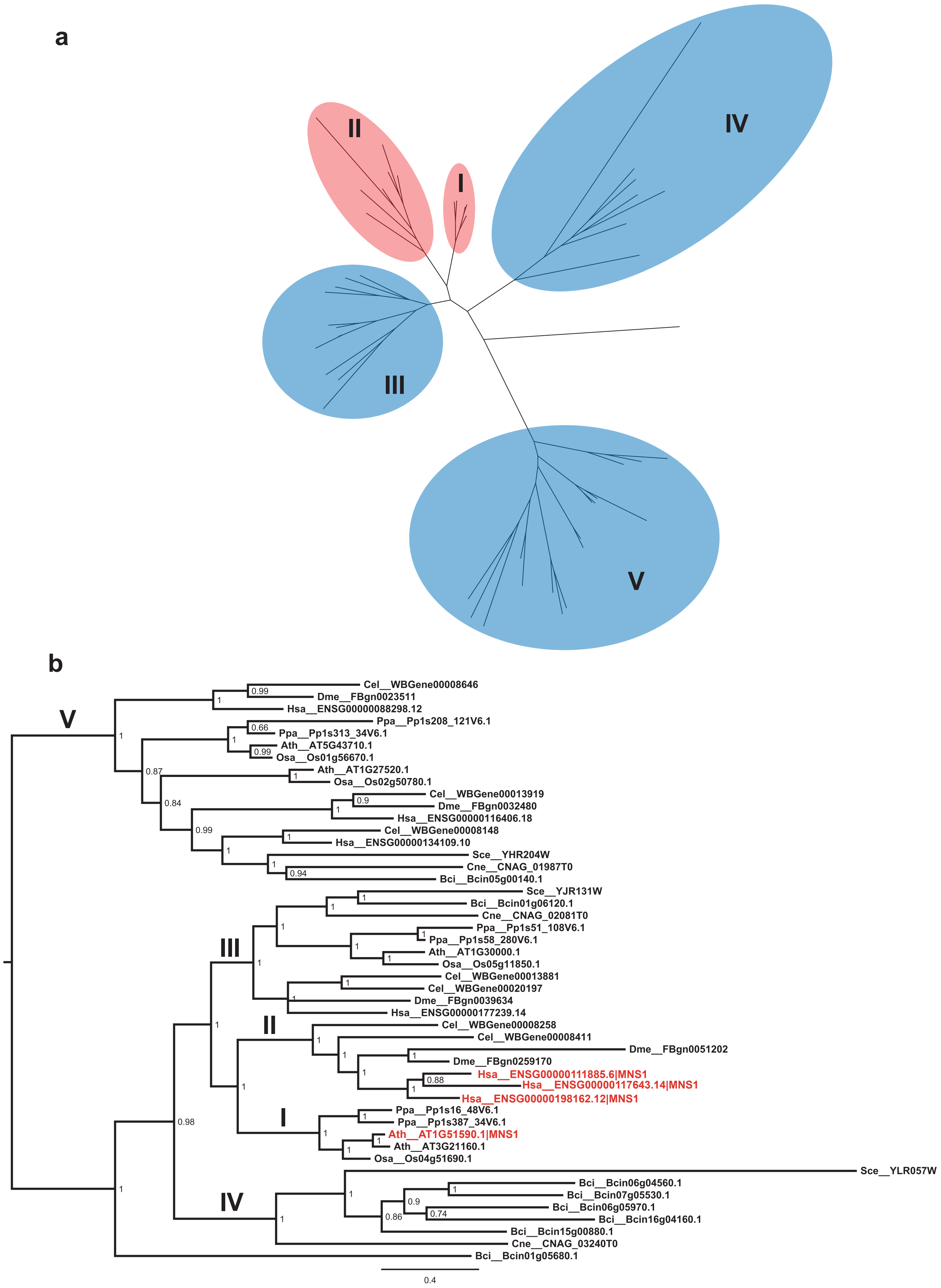

Supplementary Figure S6. Phylogeny of the genes containing domain PF01532, which include plant and animal genes encoding Golgi-α-mannosidases (in Clades I & II). a.Unrooted phylogenetic tree of the genes without tip labels. Clades I and II, where known plant and animal Mnn1genes reside, are shaded in red, while other clades are shaded in blue.b. Phylogenetic tree with gene locus IDs labeled in tips and Bayesian posterior probabilities labeled at nodes. Clade labels are in red, and also known plant and human MNS1 genes are labeled in red, with common names appending locus IDs separated by vertical lines.

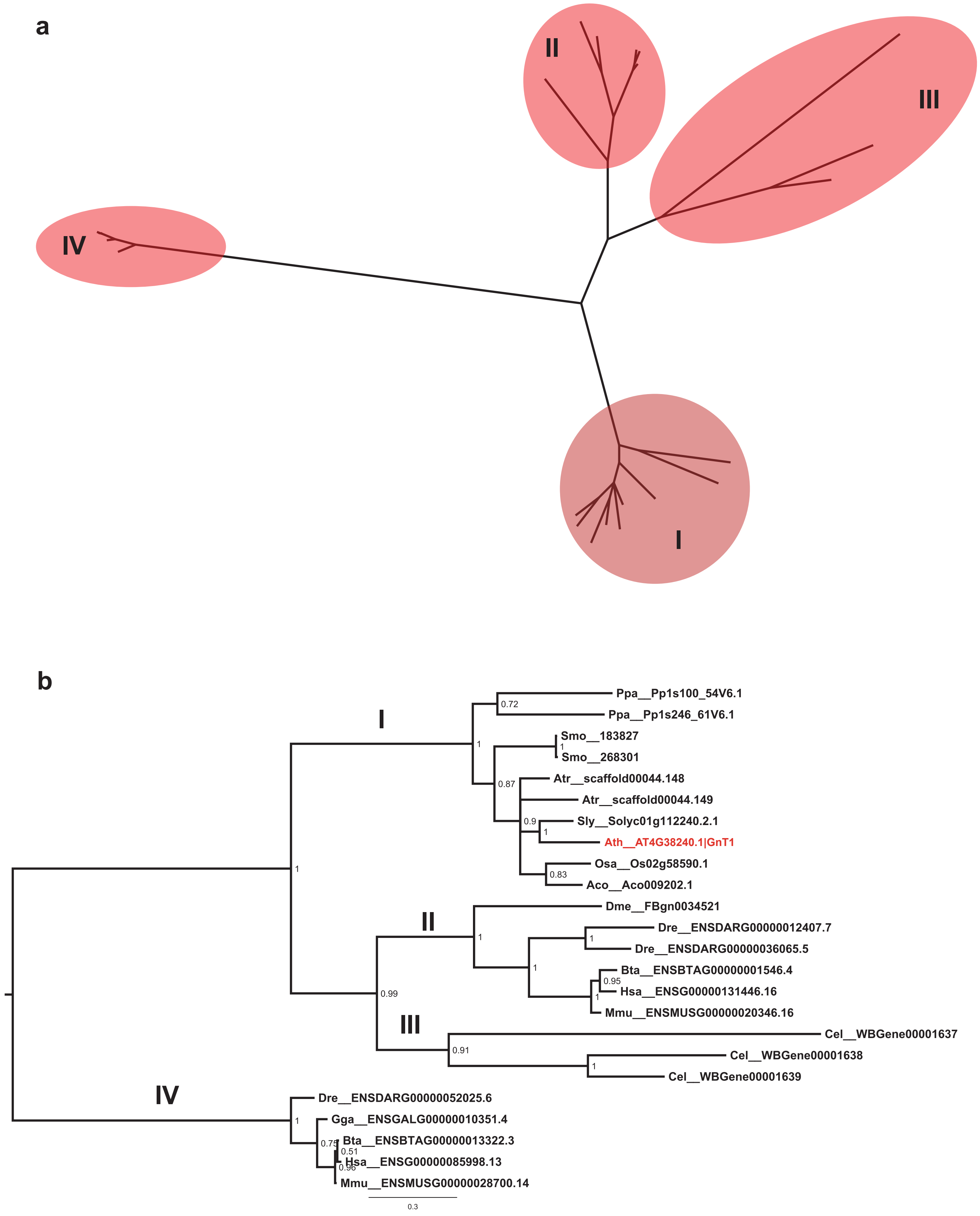

Supplementary Figure S7. Phylogeny of the genes containing domain PF03071, which include known GnTI genes. a.Unrooted phylogenetic tree of the genes without tip labels. b. Phylogenetic tree with gene locus IDs labeled in tips and Bayesian posterior probabilities labeled at nodes. the Arabidopsis gene with known functions of GnTI, AT4G38240.1, is labeled in red.

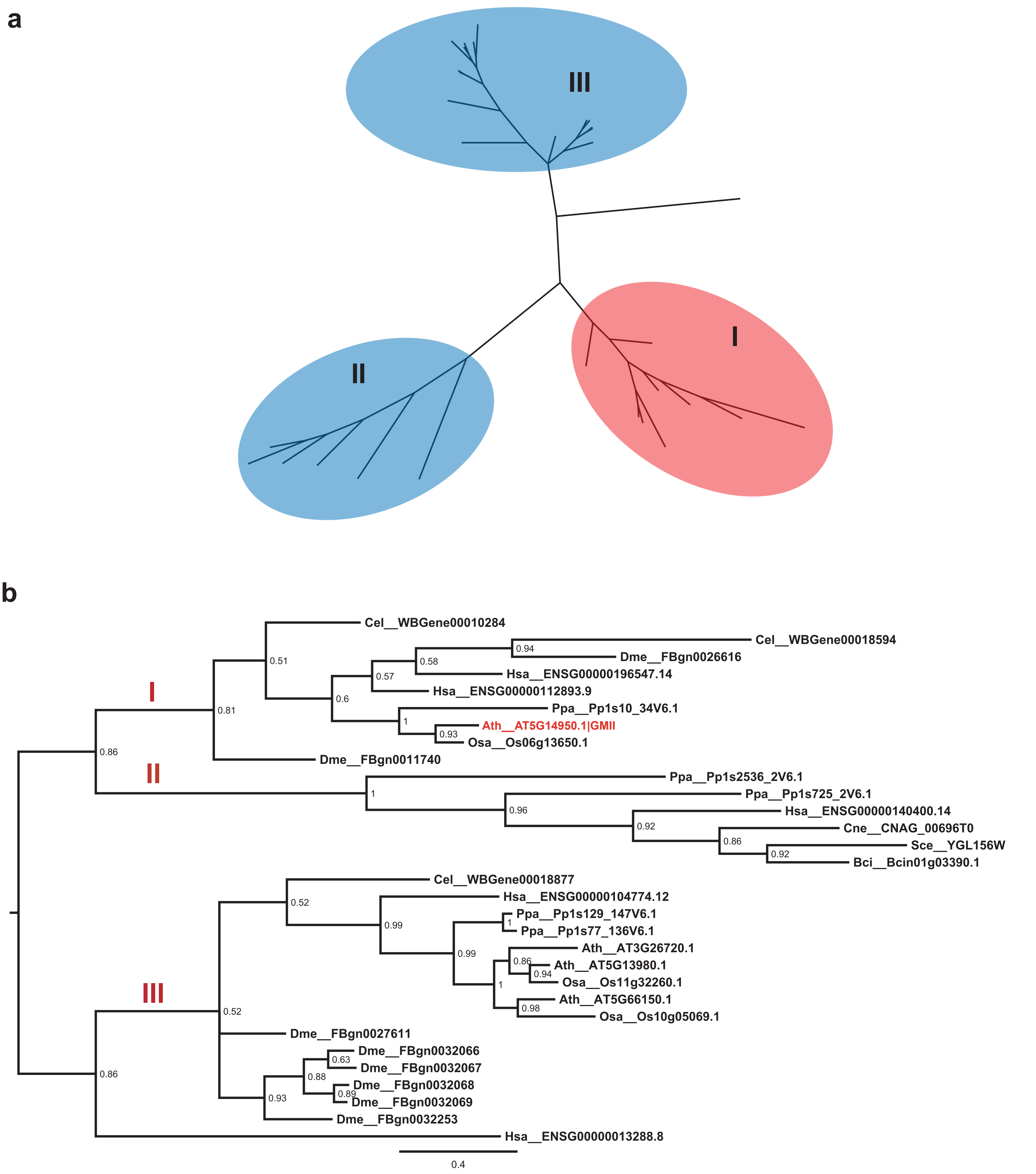

Supplementary Figure S8. Phylogeny of the genes containing domain PF09261, which include GMII genes (in Clades I). a.Unrooted phylogenetic tree of the genes without tip labels. Clde I, where Arabidopsis GMII gene is present, is shaded in red, while the other two clades are shaded in blue. b. Phylogenetic tree with gene locus IDs labeled in tips and Bayesian posterior probabilities labeled at nodes. Clade labels are in red, and also the Arabidopsis GMII gene is labeled in red, with common names appending locus IDs separated by vertical lines.

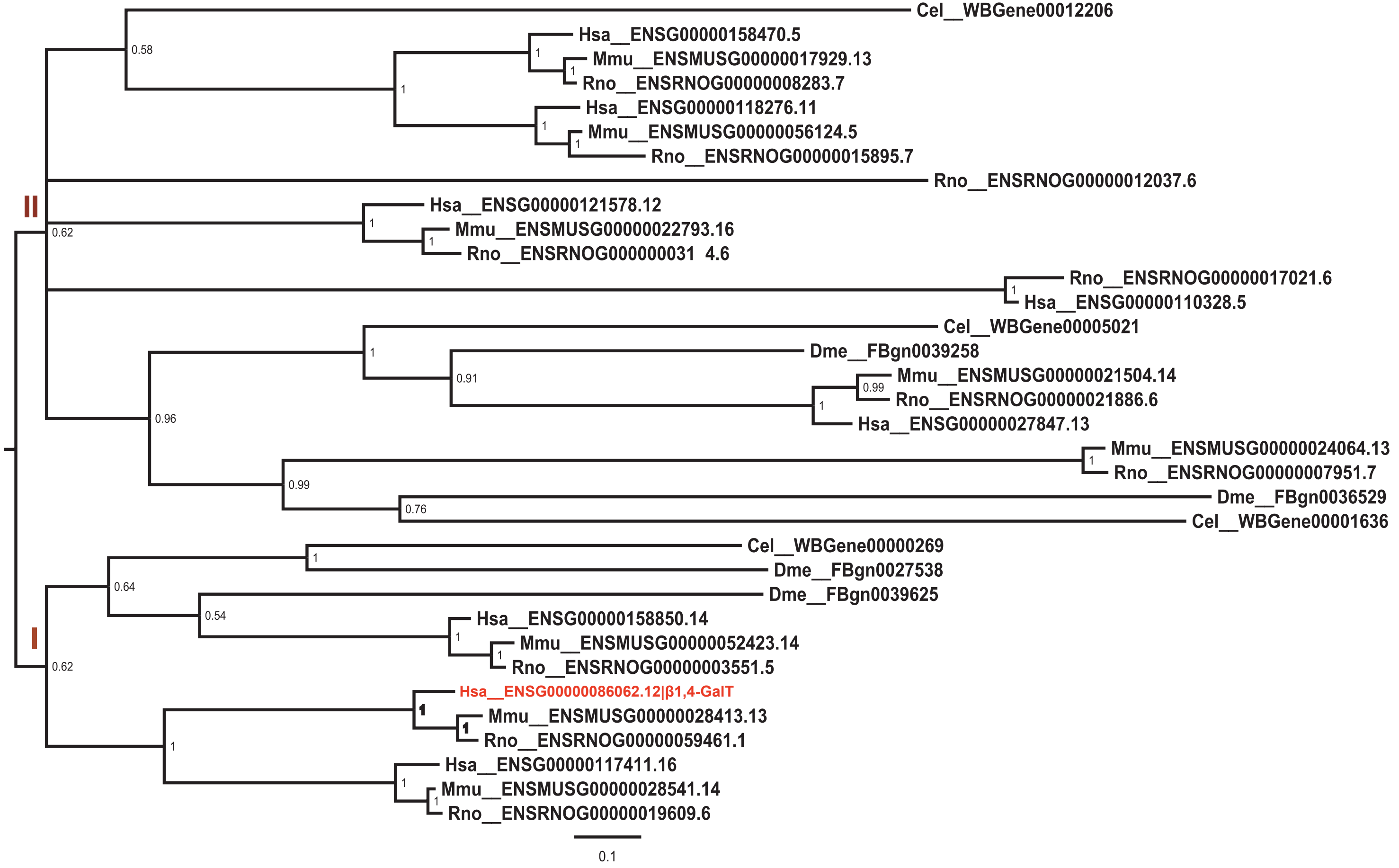

Supplementary Figure S9. Phylogeny of the genes containing domains PF13733 and PF02709, which include human β1,4-GalT gene (in Clades I, tip label is in red).

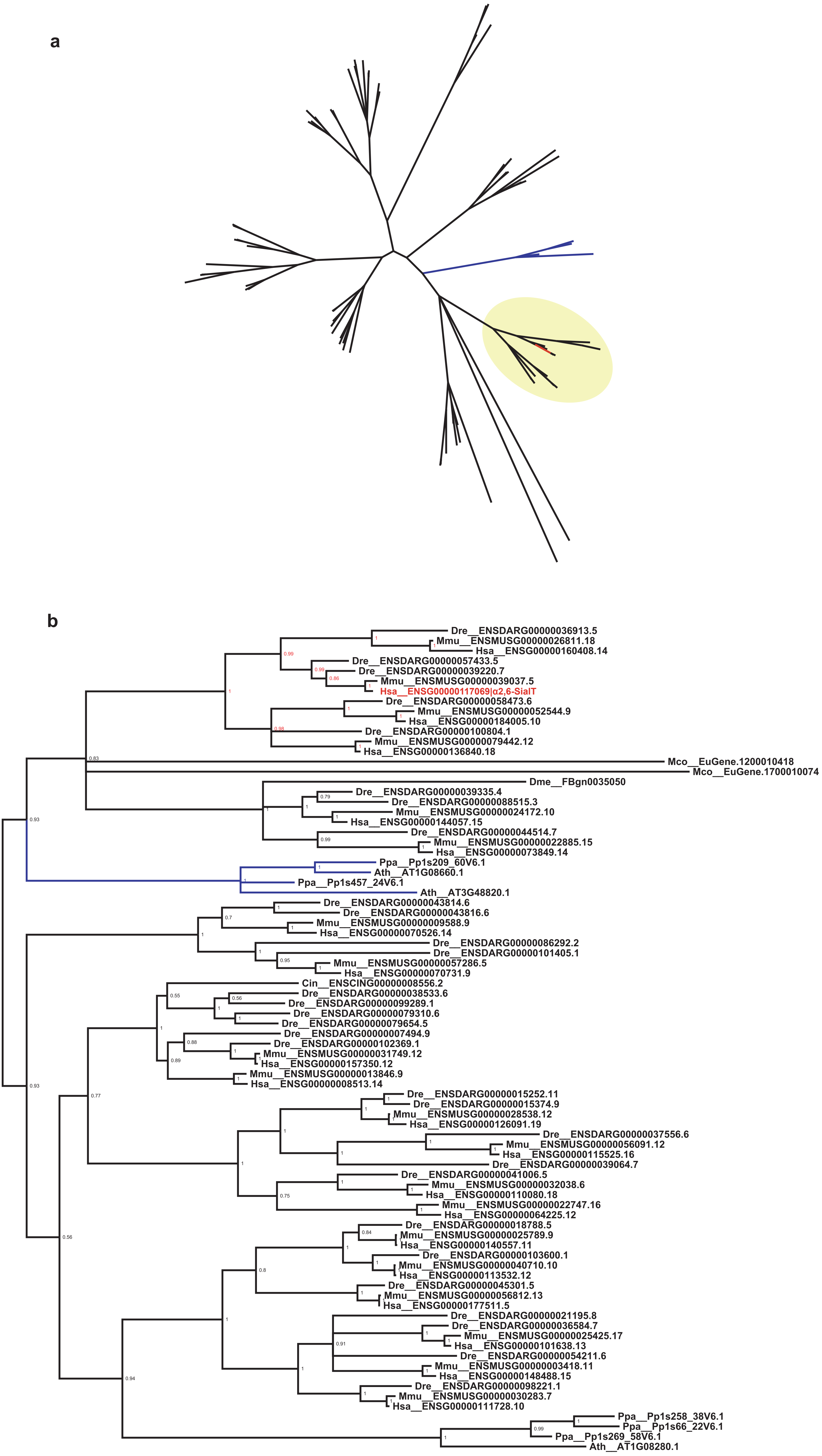

Supplementary Figure S10. Phylogeny of the genes containing domain PF00777, which include α2,6-SialT in animals. a.Unrooted phylogenetic tree of the genes without tip labels. The known human α2,6-SialT gene is labeled in red, and the clade containing this gene is shaded in yellow.The clade containing plant genes, which is close to the mammal α2,6-SialT gene, is labeled in blue. b. Phylogenetic tree with gene locus IDs labeled in tips and Bayesian posterior probabilities labeled at nodes. The known human α2,6-SialT gene is labeled in red, with common name appending locus IDs separated by vertical lines. Also, the clade containing plant genes, which is close to the mammal α2,6-SialT gene, is labeled in blue.

a

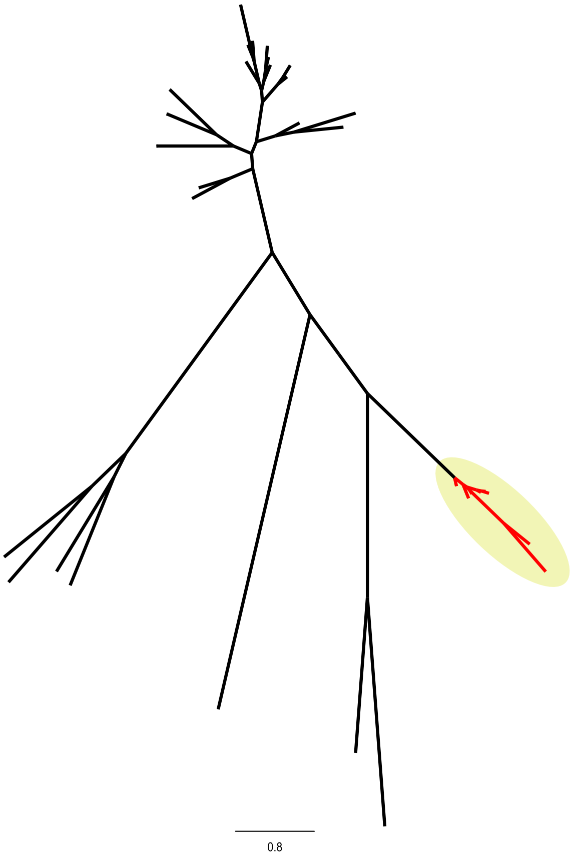

b

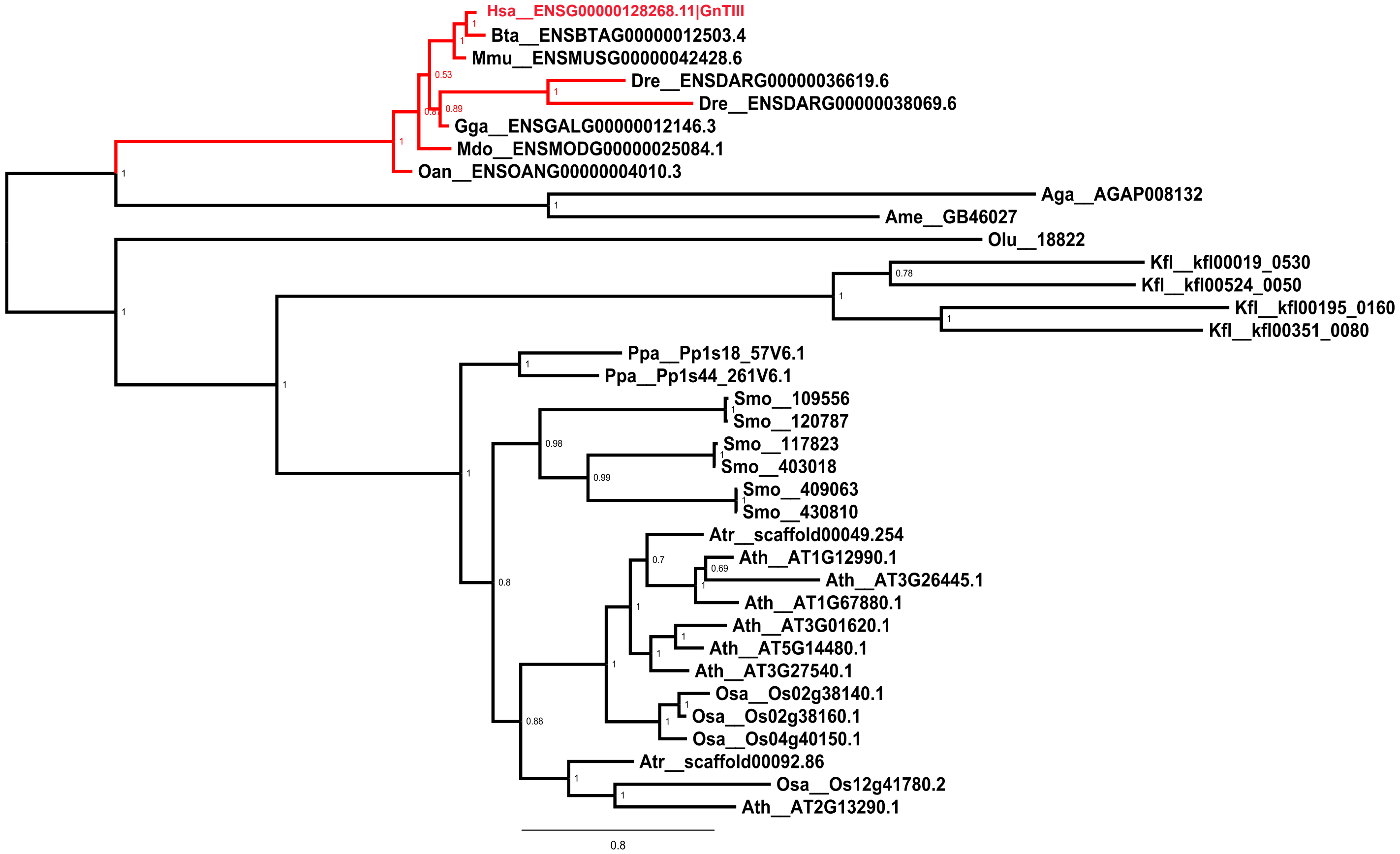

Supplementary Figure S11. Phylogeny of the genes containing domain PF04724, which include GnTIII genes in animals. a.Unrooted phylogenetic tree of the genes without tip labels. The clade containing human GnTIII gene is labeled in red, and shaded in yellow. b. Phylogenetic tree with gene locus IDs labeled in tips and Bayesian posterior probabilities labeled at nodes. The clade containing human GnTIII gene is labeled in red, and the tip representing the human GnTIII gene is labeled in red.

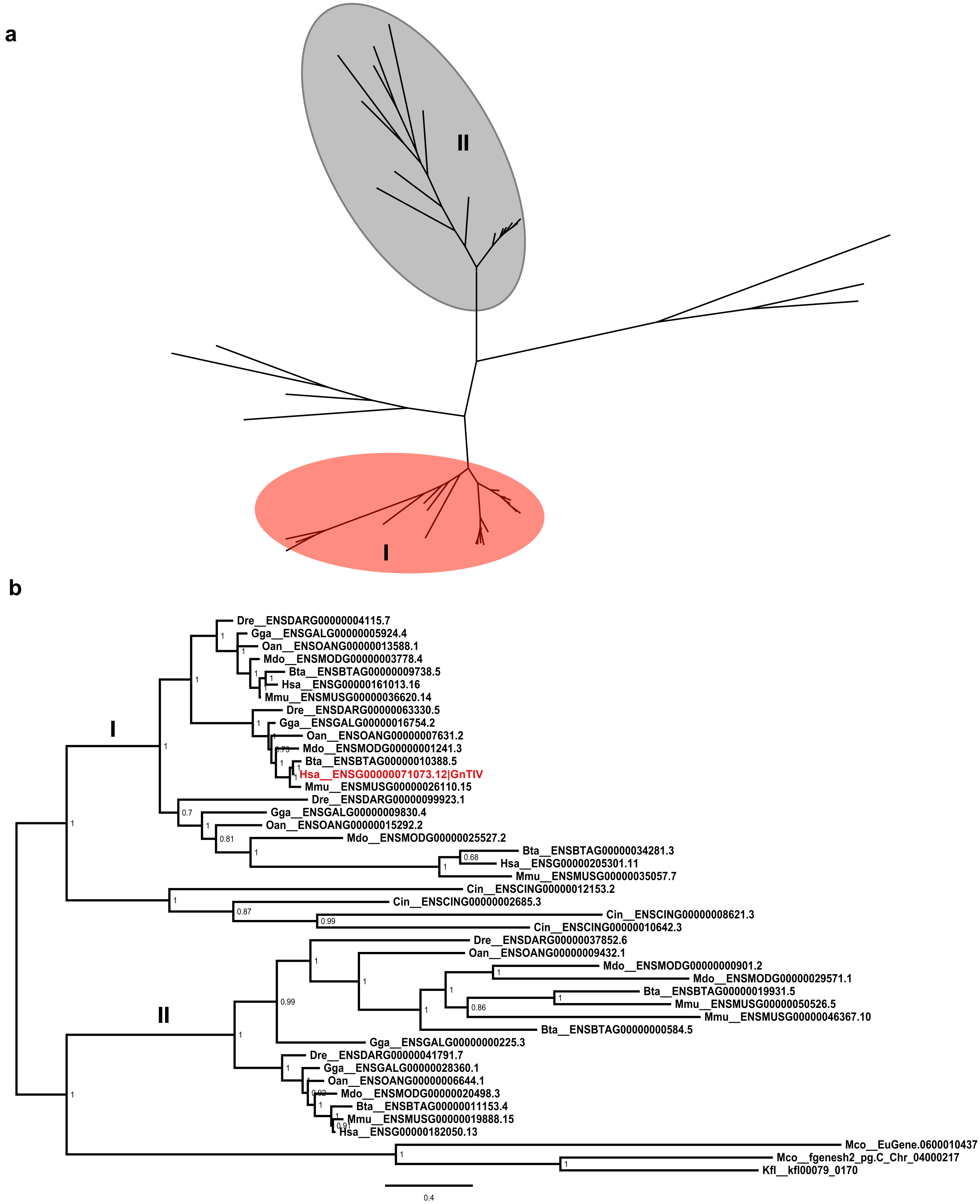

Supplementary Figure S12. Phylogeny of the genes containing domain PF04666, which include GnTIV genes in animals. a.Unrooted phylogenetic tree of the genes without tip labels. The two distincitive clades are shaded: Clade I, in which human GnTIV gene is present, is shaded in red, and Clade II is shaded in grey. b. Phylogenetic tree with gene locus IDs labeled in tips and Bayesian posterior probabilities labeled at nodes. The known human GnTIV gene is labeled in red, with common names appending locus IDs separated by vertical lines.

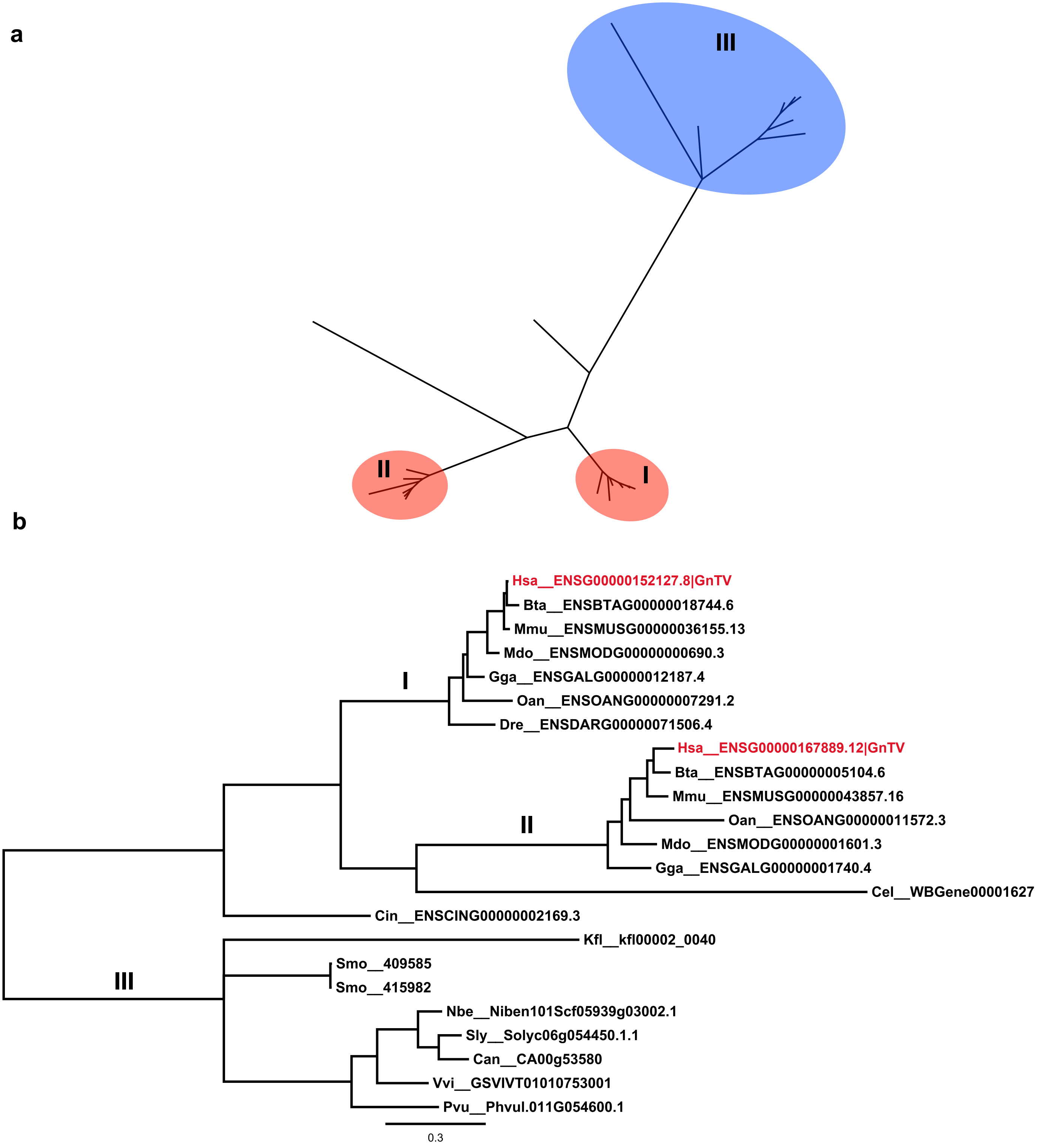

Supplementary Figure S13. Phylogeny of the genes containing domain PF15024, which include GnTV genes in animals (in Clades I&II). a.Unrooted phylogenetic tree of the genes without tip labels. Clades I &II, where the two known human GnTV genes are present, respectively, are shaded in red, while Clade III is shaded in grey. b. Phylogenetic tree with gene locus IDs labeled in tips and Bayesian posterior probabilities labeled at nodes. The two known human GnTV genes are labeled in red, with common names appending locus IDs separated by vertical lines.

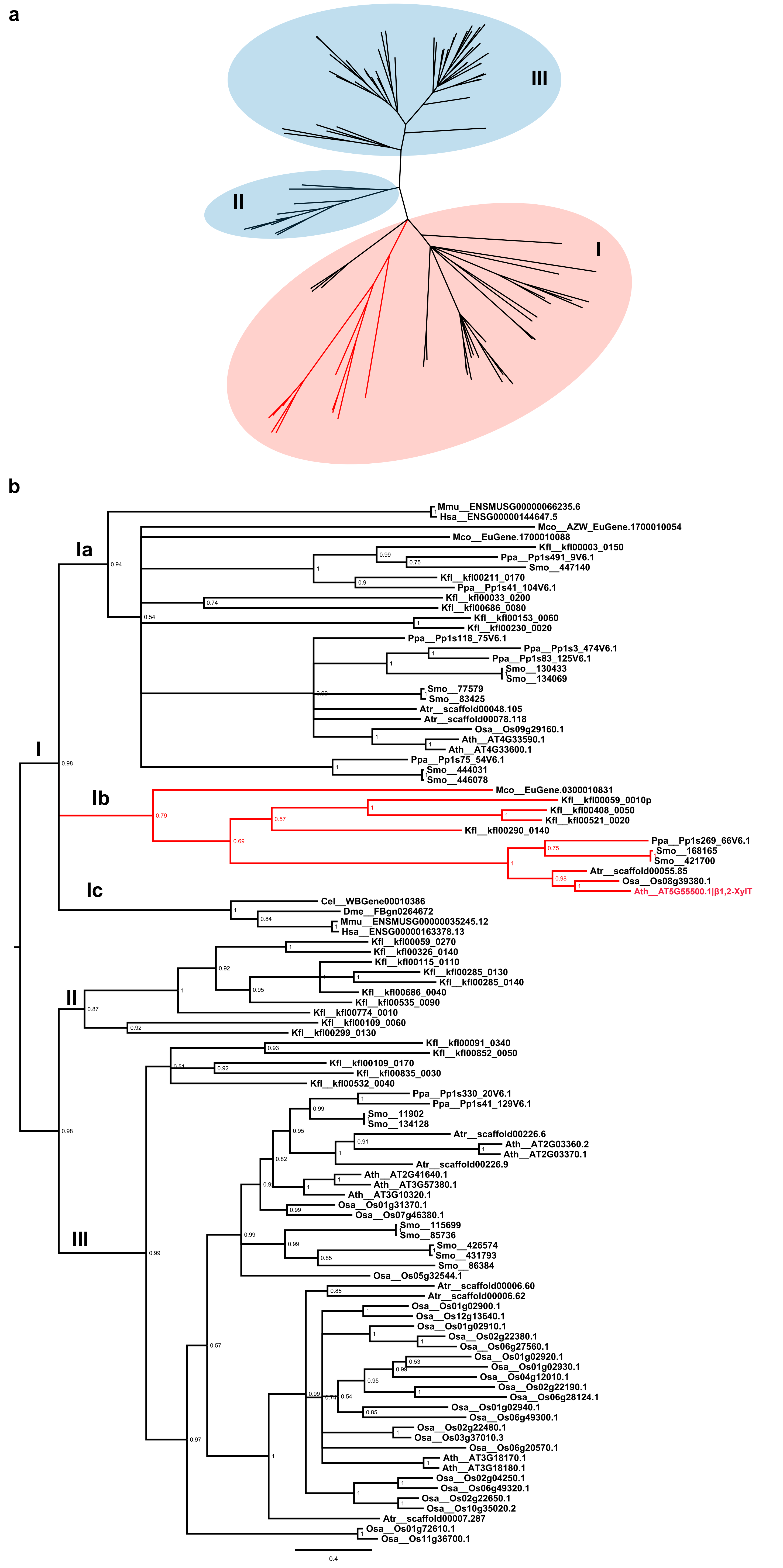

Supplementary Figure S14. Phylogeny of the genes containing domain PF04577, which include β1,2-XylT genes in plants (in Clade 1b). a.Unrooted phylogenetic tree of the genes without tip labels. Clade I, where known Arabidopsis β1,2-XylT gene is present, is shaded in red, while the other two clades are shaded in blue. The Clade 1b is labeled in red. b. Phylogenetic tree with gene locus IDs labeled in tips and Bayesian posterior probabilities labeled at nodes. Clade 1b is labeled in red, and tip label of known Arabidopsis β1,2-XylT gene is labeled in red, with common names appending locus IDs separated by vertical lines.

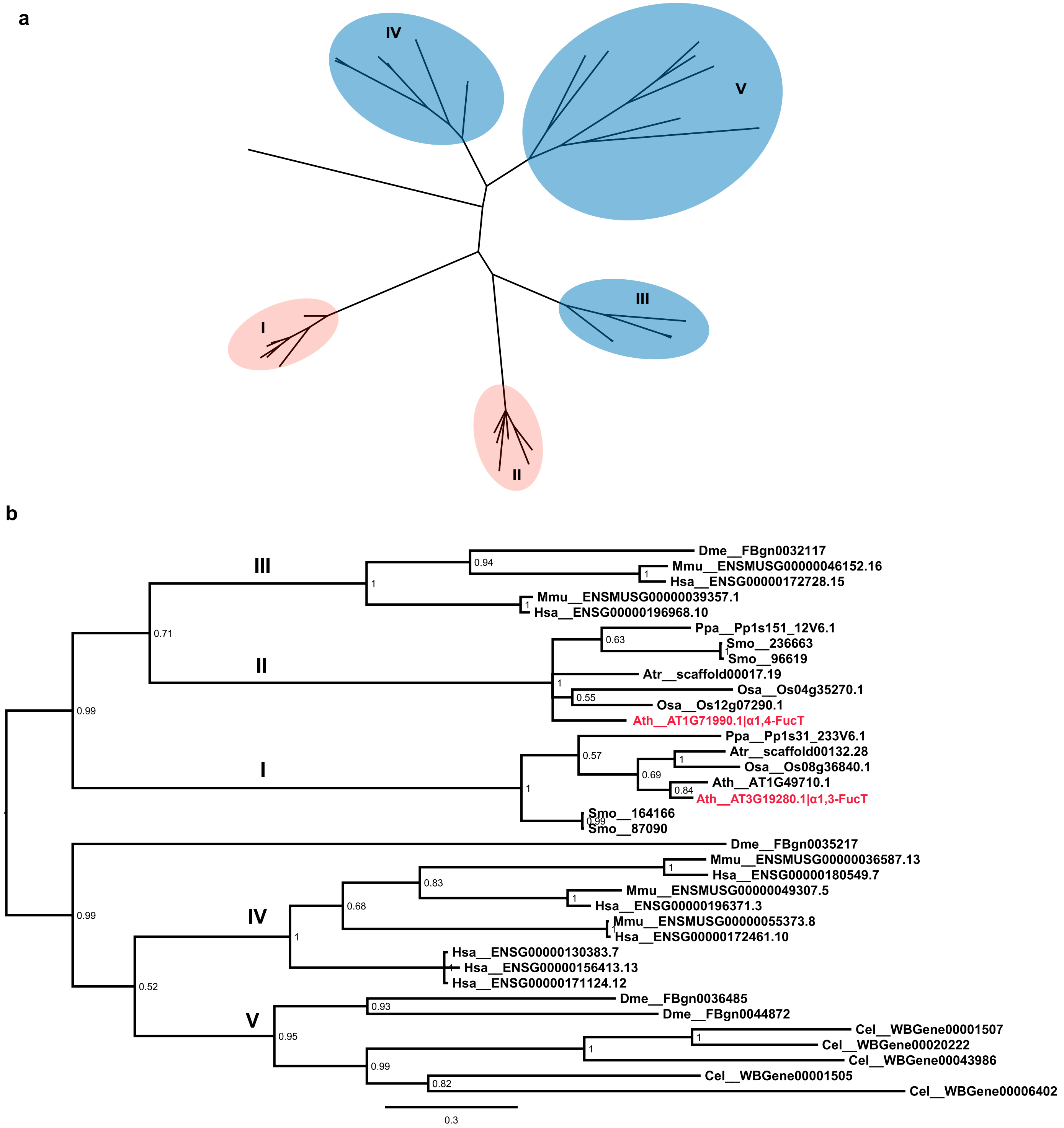

Supplementary Figure S15. Phylogeny of the genes containing domain PF00852, which include α1,3-FucT and α1,4-FucT genes in plants (in Clades I and II). a.Unrooted phylogenetic tree of the genes without tip labels. Clades I and II, where the known Arabidopsis α1,3-FucT and α1,4-FucT genes are present, respectively, are shaded in red, while other clades are shaded in blue.
b. Phylogenetic tree with gene locus IDs labeled in tips and Bayesian posterior probabilities labeled at nodes. The known α1,3-FucT and α1,4-FucT genes are highlighted in red, with common names appending locus IDs separated by vertical lines.

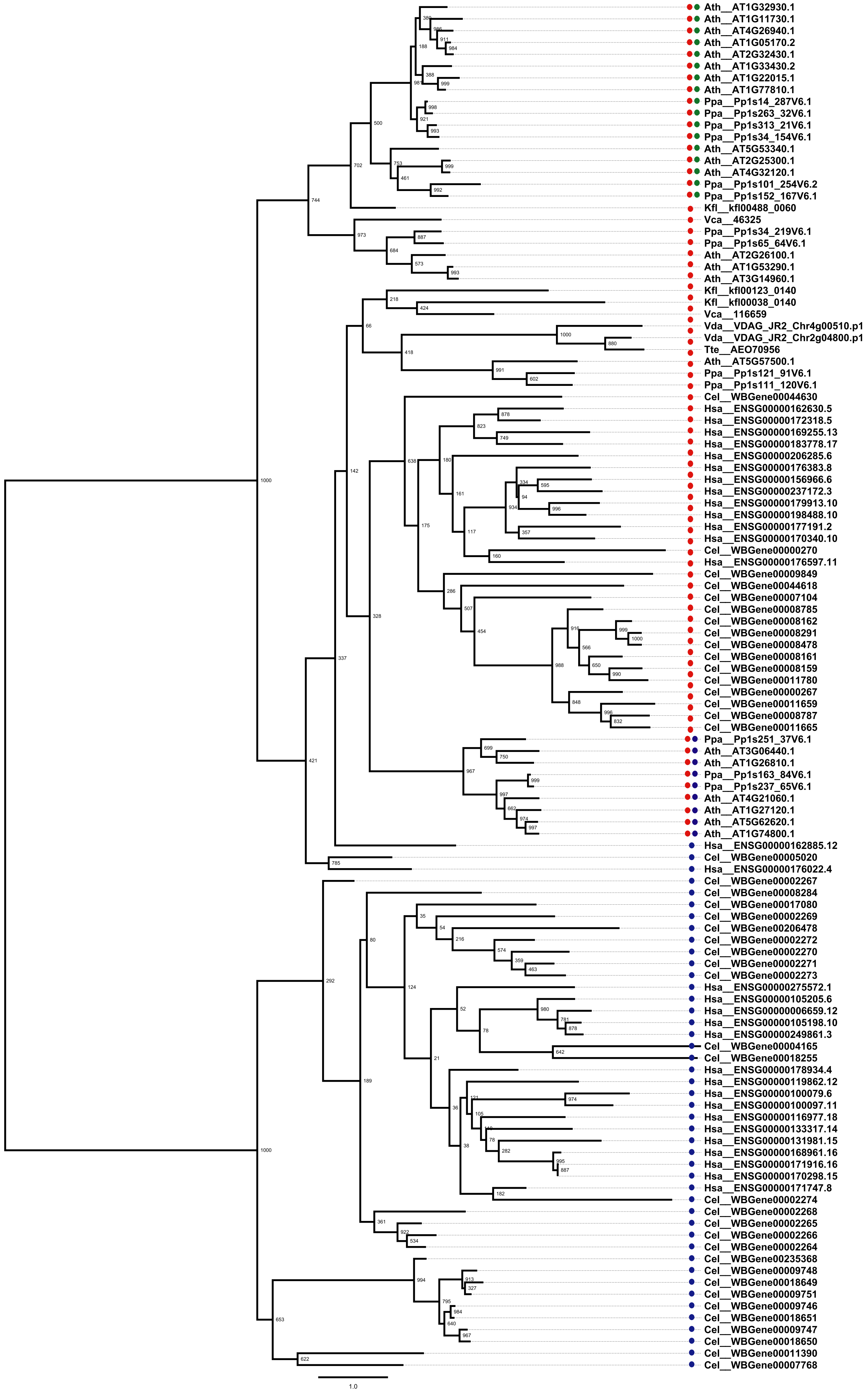

Supplementary Figure S16. Maximum likelihood phylogenetic tree of the genes containing domains PF01762 and PF00337, which include β1,3-GalT genes in plants. The tree was constructed with PhyML using WAG with 1000 bootstraps. Colored dots by every tip label represent domains the gene contain. Red dot, PF01762; green dot, PF13334; blue dot, PF00337.
